# Causal Structure Learning with Conditional and Unique Information Groups-Decomposition Inequalities

**DOI:** 10.3390/e26060440

**Published:** 2024-05-23

**Authors:** Daniel Chicharro, Julia K. Nguyen

**Affiliations:** 1 Artificial Intelligence Research Centre, Department of Computer Science, City, University of London, London EC1V 0HB, UK; 2 Department of Neurobiology, Harvard Medical School, Boston, MA 02115, USA

**Keywords:** causality, directed acyclic graphs, causal discovery, structure learning, causal structures, marginal scenarios, hidden variables, mutual information, unique information, entropic inequalities, data processing inequality, 62H22, 62D20, 94A15, 94A17

## Abstract

The causal structure of a system imposes constraints on the joint probability distribution of variables that can be generated by the system. Archetypal constraints consist of conditional independencies between variables. However, particularly in the presence of hidden variables, many causal structures are compatible with the same set of independencies inferred from the marginal distributions of observed variables. Additional constraints allow further testing for the compatibility of data with specific causal structures. An existing family of causally informative inequalities compares the information about a set of target variables contained in a collection of variables, with a sum of the information contained in different groups defined as subsets of that collection. While procedures to identify the form of these groups-decomposition inequalities have been previously derived, we substantially enlarge the applicability of the framework. We derive groups-decomposition inequalities subject to weaker independence conditions, with weaker requirements in the configuration of the groups, and additionally allowing for conditioning sets. Furthermore, we show how constraints with higher inferential power may be derived with collections that include hidden variables, and then converted into testable constraints using data processing inequalities. For this purpose, we apply the standard data processing inequality of conditional mutual information and derive an analogous property for a measure of conditional unique information recently introduced to separate redundant, synergistic, and unique contributions to the information that a set of variables has about a target.

## 1. Introduction

The inference of the underlying causal structure of a system using observational data is a fundamental question in many scientific domains. The causal structure of a system imposes constraints on the joint probability distribution of variables generated from it [1,2,3,4], and these constraints can be exploited to learn the causal structure. Causal learning algorithms based on conditional independencies [1,2,5] allow the construction of a partially oriented graph [6] that represents the equivalence class of all causal structures compatible with the set of conditional independencies present in the distribution of the observable variables (the so-called Markov equivalence class). However, without restrictions on the potential existence and structure of an unknown number of hidden variables that could account for the observed dependencies, Markov equivalence classes may encompass many causal structures compatible with the data.

Conditional independencies impose equality constraints on a joint probability distribution; namely, an independence results in the equality between conditional and unconditional probability distributions, or equivalently, in a null mutual information between independent variables. In addition to the information from independencies between the observed variables, causal information can also be obtained from other functional equality constraints [7], such as dormant independencies that would occur under active interventions [8]. Further causal inference power can be obtained incorporating assumptions on the potential form of the causal mechanisms in order to exploit additional independencies associated with hidden substructures within the generative model [9,10], or independencies related to exogenous noise terms [11,12,13]. Other approaches have studied the identifiability of specific parametric families of causal models [3,14]. However, these methods only provide additional inference power if the actual causal mechanisms conform to the required parametric form.

**Figure 1:**
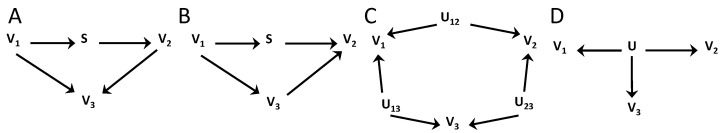
Examples of causal structures distinguishable from independencies (**A**,**B**) and structures that may only be discriminated based on inequality constraints (**C**,**D**). In this case, the structure in (**C**), and not the one in (**D**), intrinsically imposes a constraint due to dependencies between the observable variables $V_{i},i=1,2,3$ arising only from pairwise dependencies with hidden common causes.

Beyond equality constraints, the causal structure may also impose inequality constraints on the distribution of the data [15,16], which reflect non-verifiable independencies involving hidden variables. Figure 1 illustrates this distinction between pairs of causal structures distinguishable based on independence constraints (Figure 1A,B) and causal structures that may be discriminated based on inequality constraints (Figure 1C,D). The structures of Figure 1A,B belong to different Markov equivalence classes because in Figure 1A variables $V_{1}$ and $V_{2}$ are independent conditioned on *S*, while in Figure 1B, to obtain an independence it is required to further the condition on $V_{3}$. On the other hand, the structures of Figure 1C,D belong to the same equivalence class because no independencies exist between the observable variables $V_{i},i=1,2,3$. Nonetheless, if the hidden variables were also observable, these structures would be distinguishable. In Figure 1D, all the dependencies between the observable variables are caused by a single hidden variable *U*, while in Figure 1C dependencies are created pairwise by different hidden variables. In this case, a testable inequality constraint involving the observable variables reflects the non-verifiable independencies that involve also hidden variables. Intuitively, in Figure 1C, the inequality constraint imposes an upper bound on the overall degree of dependence between the three variables, given that these dependencies arise only in a pairwise manner, while in Figure 1D no such bound exists.

Importantly, unlike equality constraints, inequality constraints provide necessary but not sufficient conditions for the compatibility of data with a certain causal structure. While a certain hypothesized causal structure—like in Figure 1C—may impose the fulfillment of a given inequality intrinsically from its structure, other causal structures—like in Figure 1D—can generate data that, given a particular instantiation of the causal mechanisms, also fulfill the inequality. Accordingly, the causal inference power of inequality constraints lies in the ability to reject hypothesized causal structures that would intrinsically require the fulfillment of an inequality when that inequality is not fulfilled by the data. This means that tighter inequalities have more inferential power, giving the capacity to discard more causal structures.

Two main classes of inequality constraints have been derived. The first class corresponds to inequality constraints in the probability space, which comprise tests of compatibility such as Bell-type inequalities [17,18], instrumental inequalities [19,20], and inequalities that appear on identifiable interventional distributions [21]. The second class corresponds to inequalities involving information-theoretic quantities. The relation between these probabilistic and entropic inequalities has been examined in [22]. One approach to construct entropic inequalities combines the inequalities defining the Shannon entropic cone, i.e., associated with the non-negativity, monotonicity, and submodularity properties of entropy, and additional independence constraints related to the causal structure [23,24]. Additional causally informative inequalities can be derived if considering the so-called Non-Shannon inequalities [25,26]. When the causal structure to be tested involves hidden variables, all non-trivial entropic inequalities in the marginal scenario associated with the set of observable variables can be derived with an algorithmic procedure [23,24] that projects the set of inequalities of all variables into inequalities that only involve the subset of observable variables.

As an alternative approach, information-theoretic inequality constraints can be derived by an explicit analytical formulation [24,27]. In particular, [27] introduced inequalities comparing the information about a target variable contained in a whole collection of variables with a weighted sum of the information contained in groups of variables corresponding to subsets of the collection. Two procedures were introduced to select the composition of these groups. In a first type of inequalities, the composition of the groups is arbitrarily determined, but an inequality only exists under some conditions of independence between the chosen variables, whose fulfillment reflects the underlying causal structure. In a second type, no conditions are required for the existence of an inequality, but the groups must be ancestral sets; that is, must contain all other variables that have a causal effect on any given element of the group. In both cases, [27] showed that the coefficients in the weighted sum of the information contained in groups of variables are determined by the number of intersections between the groups.

In this work, we build upon the results of [27] and generalize their framework of groups-decomposition inequalities in several ways. First, we generalize both types of inequalities to the conditional case, when the inequalities involve conditional mutual information measures instead of unconditional ones. While this extension is trivial for the first type of inequalities, we show that for the second type it requires a definition of augmented ancestral sets. Second, we formulate more flexible conditions of independence for which the first type of inequalities exists. Third, we add flexibility to the construction of the ancestral sets that appear in the second type of inequalities. We show that, given a causal graph and a conditioning set of variables used for the conditional mutual information measures, alternative inequalities exist when determining ancestors in subgraphs that eliminate causal connections from different subsets of the conditioning variables. Furthermore, we determine conditions in which an inequality also holds when removing subsets of ancestors from the whole set of variables, hence relaxing for the second type of inequalities the requirement that the groups correspond to ancestral sets.

Apart from these generalizations, we expand the power of the approach of [27] by considering inequalities whose existence is determined by the partition into groups of a collection of variables that also contains hidden variables. That is, hidden variables can appear not only as hidden common ancestors of the collection but also as part (or even all) of the variables in the collection for which the inequality is defined. To render operational the use of inequalities derived from collections containing hidden variables, we develop procedures that allow mapping those inequalities into testable inequalities that only involve observable variables. While this mapping can be carried out by simply applying the monotonicity of mutual information to remove hidden variables from the groups, this does not work when all variables in the collection are hidden. We show that data processing inequalities [28] can be applied to obtain testable inequalities also in this case, or applied to obtain tighter inequalities than those obtained by simply removing the hidden variables. We illustrate how testable inequalities whose coefficients in the weighted sum depend on intersections among subsets of hidden variables instead of among subsets of observable variables can result into tighter inequalities with higher inferential power.

In order to derive testable groups-decomposition inequalities, we do not only apply the standard data processing inequality of conditional mutual information [28], but we derive an additional data processing inequality for the so-called *unique information* measure introduced in [29]. This measure was introduced in the framework of a decomposition of mutual information into redundant, unique, and synergistic information components [30]. Recently, alternative decompositions have been proposed to decompose the joint mutual information that a set of predictor variables has about a target variable into redundant, synergistic, and unique components [31,32,33,34,35] (among others). These alternative decompositions generally differ in the quantification of each component and differ in whether the measures fulfill certain properties or axioms. However, in our work, we do not apply the unique information measure of [29] as part of a decomposition of the joint mutual information. Instead, we show that it provides an alternative data processing inequality that holds for different causal configurations than the standard data processing inequality of conditional mutual information. In this way, the unique information data processing inequality increases the capability to eliminate hidden variables in order to obtain testable groups-decomposition inequalities. Accordingly, the groups-decomposition inequalities we derive can contain unique information terms apart from the standard mutual information and entropy measures that appear when considering the constraints of the Shannon entropic cone [23,24].

We envisage the application of the causally informative tests here proposed in the following way. Given a data set, a hypothesized causal structure is selected to test its compatibility with the data. First, the set of inequality constraints enforced by that causal structure is determined. Second, their fulfillment is evaluated from the data and the causal structure is discarded if some inequality does not hold. In the first step, the determination of the set of groups-decomposition inequalities enforced by a causal structure requires at different levels the verification of conditional independencies. This is the case, for example, with the conditional independencies that are necessary conditions for the existence of the first type of inequalities introduced by [27]. If all variables involved were observable, this verification could be conducted directly from the data. However, as mentioned above, we here consider groups-decomposition inequalities that may contain hidden variables as part of the collection of variables, which precludes this direct verification. For this reason, we will work under the assumption that statistical independencies can be assessed from the structure of the causal graph, namely with the graphical criterion of separability between nodes in the graph known as *d-separation* [36]. That is, we will rely on the assumption that graphical separability is a sufficient condition for statistical independence and hence characterize the set of groups-decomposition inequalities enforced by a causal structure without using the data. Data would only be used in the second step, in which the actual fulfillment of the inequalities is evaluated.

This paper is organized as follows. In Section 2, we review previous work relevant for our contributions. In Section 3.1, we formulate the data processing inequality for the unique information measure. In Section 3.2, we generalize the first type of inequalities of [27], formulating for the conditional case more general conditions of independence for which a groups-decomposition inequality exists. We also apply data processing inequalities to derive testable groups-decomposition inequalities when collections include hidden variables. In Section 3.3, we generalize the second type of inequalities of [27] as outlined above. In Section 4, we discuss the connection of this work with other approaches to causal structure learning and point to future continuations and potential applications. The Appendix contains proofs of the results (Appendix A and Appendix B) and a discussion of the relations between conditional independencies and d-separations required so that the inequalities here derived are applicable to test causal structures (Appendix C).

## 2. Previous Work on Information-Theoretic Measures and Causal Graphs Relevant for Our Derivations

In this section we review properties of information-theoretic measures and concepts of causal graphs relevant for our work. In Section 2.1, we review basic inequalities of the mutual information and in Section 2.2 the definition and relevant properties of the unique information measure of [29]. We then review in Section 2.3 Directed Acyclic Graphs (DAGs) and their relation to conditional independence through the graphical criterion of *d-separation* [36,37]. Finally, we review the inequalities introduced by [27] to test causal structures from information decompositions involving sums of groups of variables (Section 2.4). We do not aim to more broadly review other types of information-theoretic inequalities [23,24] also used for causal inference. The relation with these other types will be considered in the Discussion.

### 2.1. Mutual Information Inequalities Associated with Independencies

We present in Lemma 1 two well-known inequalities that will be used in our derivations. This lemma corresponds to Lemma 1 in [27]. For completion, we provide the proof of the lemma.

**Lemma 1.**  *The mutual information fulfills the following inequalities in the presence of the corresponding independencies:*

*(i) (Conditional mutual information data processing inequality): Let A, B, $B^{\prime }$, and D be four sets of variables. If $I(A;B^{\prime }|B,D)=0$, then it follows that $I\left(A;B|D\right)\geq I\left(A;B^{\prime }|D\right)$.*

*(ii) (Increase through conditioning on independent sets): Let A, B, C, and Y be four sets of variables. If I(A;C|B)=0, then I(Y;A|B)≤I(Y;A|B,C).*

**Proof.**  (i) is proven applying, in two different orders, the chain rule of the mutual information to $I(A;B,B^{\prime }|D)$:

$$I\left(A;B,B^{\prime }|D\right)=I\left(A;B|D\right)+I\left(A;B^{\prime }|B,D\right)=I\left(A;B^{\prime }|D\right)+I\left(A;B|B^{\prime },D\right).$$

Since $I(A;B^{\prime }|B,D)=0$ and the mutual information is non-negative, this implies the inequality. To prove (ii), the chain rule is applied in different orders to I(Y,C;A|B):

I(Y,C;A|B)=I(C;A|B)+I(Y;A|B,C)=I(Y;A|B)+I(C;A|B,Y).

Since I(C;A|B)=0 and the mutual information is non-negative, this implies the inequality. □

### 2.2. Definition and Properties of the Unique Information

The concept of *unique information* as part of a decomposition of the joint mutual information I(Y;X) that a set of predictor variables $X=\{X_{1},\cdots ,X_{N}\}$ has about a target (possibly multivariate) variable Y was introduced in [30]. In the simplest case of two predictors $\left\{X_{1},X_{2}\right\}$, this framework decomposes the joint mutual information about Y into four terms, namely the redundancy of $X_{1}$ and $X_{2}$, the unique information of $X_{1}$ with respect to $X_{2}$, the unique information of $X_{2}$ with respect to $X_{1}$, and the synergy between $X_{1}$ and $X_{2}$. The predictors share the redundant component, the synergistic one is only obtained by combining the predictors, and unique components are exclusive to each predictor. Several information measures have been proposed to define this decomposition, aiming to comply with a set of desirable properties which were not all fulfilled by the original proposal [29,31,32,33]. However, in this work we will not study the whole decomposition but specifically apply the bivariate measure of unique information introduced in [29]. In Section 3.1, we derive a data processing inequality for this measure and in Section 3.2 we show how it can help to obtain testable groups-decomposition inequalities for causal structures for which the standard data processing inequality of the mutual information would not allow elimination of the hidden variables. In this Section, we review the definition of the unique information measure of [29], we provide a straightforward generalization to a conditional unique information measure, and state a monotonicity property that will be used to derive the data processing inequality of the unique information. The unique information of $X_{1}$ with respect to $X_{2}$ about Y was defined as

(1) $$I(Y;X_{1}\setminus \setminus X_{2})\equiv \min_{Q\in \Delta_{P}}I_{Q}\left(Y;X_{1}|X_{2}\right),$$

where $\Delta_{P}$ is defined as the set of distributions on $\left\{Y,X_{1},X_{2}\right\}$ that preserve the marginals $P(Y,X_{1})$ and $P(Y,X_{2})$ of the original distribution $P(Y,X_{1},X_{2})$. The notation $I_{Q}$ is used to indicate that the mutual information is calculated on the probability distribution *Q*. We use $I(Y;X_{1}\setminus \setminus X_{2})$ to refer to the unique information of $X_{1}$ with respect to $X_{2}$, compared to $I(Y;X_{1}|X_{2})$, which is the standard conditional information of $X_{1}$ given $X_{2}$. We use the notation $X_{1}\setminus \setminus X_{2}$ instead of the notation $X_{1}\setminus X_{2}$ introduced by [29] to differentiate it from the set notation $X_{1}\setminus X_{2}$, which indicates the subset of variables in $X_{1}$ that is not contained in $X_{2}$, since we will also be using this set notation. This unique information measure is a maximum entropy measure, since all distributions within $\Delta_{P}$ preserve the conditional entropy $H(Y|X_{2})$, and hence the minimization is equivalent to a maximization of the conditional entropy $H(Y|X_{1},X_{2})$. The rationale that supports this definition is that the unique information of $X_{1}$ with respect to $X_{2}$ about Y has to be determined by the marginal probabilities $P(Y,X_{1})$ and $P(Y,X_{2})$, and cannot depend on any additional structure in the joint distribution that contributes to the dependence between $\left\{X_{1},X_{2}\right\}$ and Y [29]. This additional contribution is removed by minimizing within $\Delta_{P}$.

In a straightforward generalization, we define the conditional unique information given another set of variables Z as

(2) $$I(Y;X_{1}\setminus \setminus X_{2}\left|Z\right)\equiv \min_{Q\in \Delta_{P^{\prime }}}I_{Q}\left(Y;X_{1}|X_{2},Z\right),$$

where $\Delta_{P^{\prime }}$ is the set of distributions on $\left\{Y,X_{1},X_{2},Z\right\}$ that preserve the marginals $P(Y,X_{1},Z)$ and $P(Y,X_{2},Z)$ of the original $P(Y,X_{1},X_{2},Z)$. By construction [29], the conditional unique information is bounded as

(3) $$\min\left\{I\left(Y;X_{1}|Z\right),I\left(Y;X_{1}|X_{2},Z\right)\right\}\geq I(Y;X_{1}\setminus \setminus X_{2}\left|Z\right)\geq 0.$$

This is consistent with the intuition of the decomposition that the unique information is a component exclusive of $X_{1}$. In Lemma 2, we present a type of monotonicity fulfilled by the conditional unique information. This result is a straightforward extension to the conditional case of the one stated in Lemma 3 of [38]. We include the full proof because it will be useful in the Results section to prove a related data processing inequality for the unique information. To better suit our subsequent use of notation, we consider the two predictors to be $Z_{1}$ and $\left\{X_{1},X_{1}^{\prime }\right\}$, and the conditioning set to be $Z_{2}$.

**Lemma 2.**  *The maximum entropy conditional unique information is monotonic on its second argument, corresponding to the non-conditioning predictor, as follows:*

$$I(Y;X_{1},X_{1}^{\prime }\setminus \setminus Z_{1}|Z_{2})\geq I(Y;X_{1}\setminus \setminus Z_{1}\left|Z_{2}\right).$$

**Proof.**  Consider the distribution $P_{1,1^{\prime }}\equiv P\left(Y,X_{1},X_{1}^{\prime },Z_{1},Z_{2}\right)$ and its marginal $P_{1}\equiv P\left(Y,X_{1},Z_{1},Z_{2}\right)$. Consider any distribution $Q_{1,1^{\prime }}\in \Delta_{P_{1,1^{\prime }}}$ and its marginal $Q_{1}$ on $\left(Y,X_{1},Z_{1},Z_{2}\right)$. Then $Q_{1}\in \Delta_{P_{1}}$. By monotonicity of the mutual information, $I_{Q_{1,1^{\prime }}}\left(Y;X_{1}|Z_{1},Z_{2}\right)$ is lower than or equal to $I_{Q_{1,1^{\prime }}}\left(Y;X_{1},X_{1}^{\prime }|Z_{1},Z_{2}\right)$. Since $I_{Q_{1,1^{\prime }}}\left(Y;X_{1}|Z_{1},Z_{2}\right)$ does not have $X_{1}^{\prime }$ as an argument, it is equal to the information calculated on its marginal $I_{Q_{1}}\left(Y;X_{1}|Z_{1},Z_{2}\right)$. Since this holds for any distribution in $\Delta_{P_{1,1^{\prime }}}$, it holds in particular for the distribution $Q_{1,1^{\prime }}^{*}$ that minimizes $I(Y;X_{1},X_{1}^{\prime }|Z_{1},Z_{2})$ in $\Delta_{P_{1,1^{\prime }}}$. Since $Q_{1}^{*}$ belongs to $\Delta_{P_{1}}$, the minimum of $I(Y;X_{1}|Z_{1},Z_{2})$ in $\Delta_{P_{1}}$ is equal to or smaller than $I_{Q_{1}^{*}}\left(Y;X_{1}|Z_{1},Z_{2}\right)$ and hence equal to or smaller than $I_{Q_{1,1^{\prime }}^{*}}\left(Y;X_{1},X_{1}^{\prime }|Z_{1},Z_{2}\right)$. □

### 2.3. Causal Graphs and Conditional Independencies

We here review basic notions of Directed Acyclic Graphs (DAGs) and the relation between causal structures and dependencies. Consider a set of random variables $V=\{V_{1},\cdots ,V_{n}\}$. A DAG G=(V;E) consists of nodes V and edges E between the nodes. The graph contains $V_{i}\to V_{j}$ for each $(V_{i};V_{j})\in E$. We refer to *V* as both a variable and its corresponding node. Causal influences can be represented in acyclic graphs given that causal mechanisms are not instantaneous and causal loops can be spanned using separate time-indexed variables. A path in *G* is a sequence of (at least two) distinct nodes $V_{1},\cdots ,V_{m},$ such that there is an edge between $V_{k}$ and $V_{k+1}$ for all k=1,⋯,m−1. If all edges are directed as $V_{k}\to V_{k+1}$ the path is a causal or directed path. A node $V_{i}$ is a collider in a path if it has incoming arrows $V_{i-1}\to V_{i}\leftarrow V_{i+1}$ and is a noncollider otherwise. A node $V_{i}$ is called a parent of $V_{j}$ if there is an arrow $V_{i}\to V_{j}$. The set of parents is denoted ${\mathrm{Pa}}_{V_{j}}$. A node $V_{i}$ is called an ancestor of $V_{j}$ if there is a directed path from $V_{i}$ to $V_{j}$. Conversely, in this case $V_{j}$ is a descendant of $V_{i}$. For convenience, we define the set of ancestors $an_{G}\left(V_{i}\right)$ as including $V_{i}$ itself, and the set of descendants $D_{G}\left(V_{i}\right)$ as also containing $V_{i}$ itself.

The link between generative mechanisms and causal graphs relies on the fact that in the graph a variable $V_{i}$ is a parent of another variable $V_{j}$ if and only if it is an argument of an underlying functional equation that captures the mechanisms that generate $V_{j}$; that is, an argument of $V_{j}:=f_{V_{j}}\left({\mathrm{Pa}}_{V_{j}},\varepsilon_{V_{j}}\right)$, where $\varepsilon_{V_{j}}$ captures additional sources of stochasticity exogenous to the system. If a DAG constitutes an accurate representation of the causal mechanisms, an isomorphic relation exists between the conditional independencies that hold between variables in the system and a graphical criterion of separability between the nodes, called *d-separation* [36]. Two nodes *X* and *Y* are *d-separated* given a set of nodes S if and only if no S-active paths exist between *X* and *Y*. A path is active given the conditioning set S (S-active) if no noncollider in the path belongs to S and every collider in the path either is in S or has a descendant in S. A causal structure *G* and a generated probability distribution p(V) are *faithful*[1,2] to one another when a conditional independence between *X* and *Y* given S—denoted by $X{⊥}_{P}Y|S$—holds if and only if there is no S-active path between them; that is, if *X* and *Y* are d-separated given S—denoted by $X{⊥}_{G}Y|S$. Accordingly, faithfulness is assumed in the algorithms of causal inference [1,2] that examine conditional independencies to characterize the Markov equivalence class of causal structures that share a common set of independencies. A well-known example of a system that is unfaithful to its causal structure is the exclusive-OR (X-OR) logic gate, whose output is independent of the two inputs separately but dependent on them jointly.

In contrast to the algorithms that infer Markov equivalence classes, we will show that the applicability of the groups-decomposition inequalities here studied relies on the assumption that d-separability is a sufficient condition for conditional independence. That is, instead of an *if and only if* relation between d-separability and conditional independence, as required in the faithfulness assumption, it is enough to assume that d-separability implies conditional independence. As we further discuss in Appendix C, this is a substantially weaker assumption, since usually faithfulness is violated due to the presence of independencies that are incompatible with the causal structure. This is the case, for example, of the X-OR logic gate, for which faithfulness is violated because the inputs are separately independent of the output despite each having an arrow towards the output in the corresponding causal graph. Conversely, the X-OR gate complies with d-separability being a sufficient condition for independence, since in the graph only the input nodes are d-separated and the corresponding input variables of the X-OR gate are independent. Despite only requiring that d-separability implies independence, to simplify the presentation of our results in the main text we will assume faithfulness and indistinctively use X⊥Y|S to indicate statistical independence and graphical separability, instead of distinguishing between $X{⊥}_{P}Y|S$ and $X{⊥}_{G}Y|S$. In Appendix C, we will more closely examine how in the proofs of our results the sufficient condition of d-separability for conditional independencies is enough. An important implication of independencies following from d-separability is that, if variables $\left\{X_{1},X_{2}\right\}$ are separately independent from *Y*—namely $Y⊥X_{1}$ and $Y⊥X_{2}$—because of the lack of any connection between node *Y* and both nodes $X_{1}$ and $X_{2}$, then $\left\{X_{1},X_{2}\right\}$ cannot be jointly dependent on *Y*, namely Y 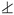 $\left\{X_{1},X_{2}\right\}$ cannot occur. This is because d-separability between node *Y* and the set of nodes $\left\{X_{1},X_{2}\right\}$ is determined by separately considering the lack of active paths between *Y* and each node $X_{1}$ and $X_{2}$. Since the set of paths between *Y* and $\left\{X_{1},X_{2}\right\}$ is the union of the paths between *Y* and both $X_{1}$ and $X_{2}$, considering $\left\{X_{1},X_{2}\right\}$ jointly does not add new paths that could create a dependence of *Y* with $\left\{X_{1},X_{2}\right\}$. A dependence can only be created by conditioning on some other variable, which could activate additional paths by activating a collider.

### 2.4. Inequalities for Sums of Information Terms from Groups of Variables

We now review two results in [27] that are at the foundation of our results. The first corresponds to their Proposition 1. We provide a slightly more general formulation that is useful for subsequent extensions.

**Proposition 1.**  *(Decomposition of information from groups with conditionally independent non-shared components): Consider a collection of groups $A_{\left[n\right]}\equiv \left\{A_{1},\cdots ,A_{n}\right\}$, where each group $A_{i}$ consists of a subset of observable variables $A_{i}\subset O$, being O the set of all observable variables. For every $A_{i}\in A_{\left[n\right]}$, define $d_{i}$ as the maximal value such that $A_{i}$ has a non-empty intersection where it intersects jointly with $d_{i}-1$ other distinct groups out of $A_{\left[n\right]}$. Consider a conditioning set Z and target variables Y. If each group is conditionally independent given Z from the non-shared variables in each other group (i.e., $A_{i}⊥A_{j}\setminus A_{i}|Z,\forall i,j$), then the conditional information that $A_{\left[n\right]}$ has about the target variables Y given Z is bounded from below by*

$$I\left(Y;A_{\left[n\right]}|Z\right)\geq \sum_{i=1}^{n}\frac{1}{d_{i}}I\left(Y;A_{i}|Z\right).$$

**Proof.**  The proof is presented in Appendix A. It is a generalization to the conditional case of the proof of Proposition 1 in [27] and a slight generalization that allows for dependencies to exist between variables shared by two groups as long as dependencies with non-shared variables do not exist. □

An illustration of Proposition 1 for the unconditional case is presented in Figure 3 of [27], together with further discussion. In Section 3.2 we will provide further illustrations for the extensions of Proposition 1 that we introduce. We will use $d\equiv \{d_{1},\cdots ,d_{n}\}$ to indicate the maximal values for all groups. We will add a subindex $d_{A_{\left[n\right]}}$ to specify the collection if different collections are compared. A trivial refinement of Proposition 1 would consider $I(Y;A_{\left[n\right]}\setminus Z|Z)$ and for each group $I(Y;A_{i}\setminus Z|Z)$. This may lead to a tighter lower bound by decreasing some values in d if some intersections between groups occur in Z. We do not present this refinement in order to simplify the presentation.

The second result from [27] that we will be relying on is their Theorem 1. We present a version that is slightly reduced and modified, which is more convenient in order to relate to our own results.

**Theorem 1.**  *(Decomposition of information in ancestral groups.) Let G be a DAG model that includes nodes corresponding to the variables in a collection of groups $A_{\left[n\right]}\equiv \left\{A_{1},\cdots ,A_{n}\right\}$, which is a subset all observable variables O. Let $an_{G}\left(A_{\left[n\right]}\right)\equiv \left\{an_{G}\left(A_{1}\right),\cdots ,an_{G}\left(A_{n}\right)\right\}$ be the collection of ancestors of the groups, as determined by G. For every ancestral set of a group, $an_{G}\left(A_{i}\right)$, let $d_{i}\left(G\right)$ be maximal, such that there is a non-empty joint intersection of $an_{G}\left(A_{i}\right)$ and other $d_{i}\left(G\right)-1$ distinct ancestral sets out of $an_{G}\left(A_{\left[n\right]}\right)$. Let Y be a set of target variables. Then the information of $an_{G}\left(A_{\left[n\right]}\right)$ about Y is bounded as*

$$H\left(Y\right)\geq I\left(Y;an_{G}\left(A_{\left[n\right]}\right)\right)\geq \sum_{i=1}^{n}\frac{1}{d_{i}\left(G\right)}I\left(Y;an_{G}\left(A_{i}\right)\right).$$

**Proof.**  The original proof can be found in [27]. □

In contrast to Proposition 1, a generalization to the conditional mutual information is not trivial and will be developed in Section 3.3. We will also propose additional generalizations regarding which graph to use to construct the ancestral sets and conditions to exclude some ancestors from the groups. In their work, [27] conceptualized Y as corresponding to leaf nodes in the graph, for example providing some noisy measurement of $A_{\left[n\right]}$, with $Y=A_{\left[n\right]}$ being the case of perfect measurement. While this conceptualization guided their presentation, their results were general, and here we will not assume any concrete causal relation between Y and $A_{\left[n\right]}$. We have slightly modified the presentation of Theorem 1 from [27] to add the upper bound and to remove some additional subcases with extra assumptions presented in their work. The upper bound is the standard upper bound of mutual information by entropy [28]. In the Results, we will also be interested in cases in which $an_{G}\left(A_{\left[n\right]}\right)$ contains hidden variables, so that $I(Y;an_{G}\left(A_{\left[n\right]}\right))$ cannot be estimated. Given the monotonicity of mutual information, the terms from each ancestral group can be lower bounded by the information in the observable variables within each group and H(Y) is used as a testable upper bound.

There are two main differences between Proposition 1 and Theorem 1. First, Theorem 1 does not impose conditions of independence for the inequality to hold. Second, while the value $d_{i}$ of each group $A_{i}$ is determined in Proposition 1 by the overlap between groups, with no influence of the causal structure relating the variables, on the other hand in Theorem 1 the value $d_{i}\left(G\right)$ depends on the causal structure, since it is determined from the intersections between ancestral sets. Despite these differences, given the relation between causal structure and independencies reviewed in Section 2.3, both types of inequalities can have causal inference power to test the compatibility of certain causal structures with data.

## 3. Results

In Section 3.1, we introduce a data processing inequality for the conditional unique information measure of [29]. In Section 3.2, we develop new information inequalities involving groups of variables and examine how data processing inequalities can help to derive testable inequalities in the presence of hidden variables. In Section 3.3, we develop new information inequalities involving ancestral sets. The application of these inequalities for causal structure learning is discussed. As justified in the proofs of our results (Appendix A and Appendix B) and further discussed in Appendix C, our derivations of groups-decomposition inequalities only rely on the assumption that d-separability implies conditional independence. No further assumptions are used in our work, in particular, our application of the unique information measures of [29] does not require any assumption regarding the precise distribution of the joint mutual information among redundancy, unique, and synergistic components.

### 3.1. Data Processing Inequality for Conditional Unique Information

**Proposition 2.**  *(Conditional unique information data processing inequality): Let A, B, $B^{\prime }$, D, and E be five sets of variables. If $I(A;B^{\prime }|B,E)=0$, then $I(A;B,B^{\prime }\setminus \setminus D|E)=I(A;B\setminus \setminus D|E)\geq I(A;B^{\prime }\setminus \setminus D|E)$.*

**Proof.**  Let $P_{BB^{\prime }}\equiv P\left(A,B,B^{\prime },D,E\right)$ be the original distribution of the variables and define $\Delta_{P_{BB^{\prime }}}$ as the set of distributions on $\left\{A,B,B^{\prime },D,E\right\}$ that preserve the two marginals $P(A,B,B^{\prime },E)$ and P(A,D,E). Let $P_{B}\equiv P\left(A,B,D,E\right)$ be the marginal of $P_{BB^{\prime }}$ and $\Delta_{P_{B}}$ be the set of distributions that preserve the marginals P(A,B,E) and P(A,D,E). By the definition of unique information (Equation (2))

(4) $$\begin{array}{cc} \; & I(A;B,B^{\prime }\setminus \setminus D|E)\equiv \min_{Q_{BB^{\prime }}\in \Delta_{P_{BB^{\prime }}}}I_{Q_{BB^{\prime }}}\left(A;B,B^{\prime }|D,E\right)\overset{\left(a\right)}{=}\\ \; & \min_{Q_{BB^{\prime }}\in \Delta_{P_{BB^{\prime }}}}\left[I_{Q_{BB^{\prime }}}\left(A;B|D,E\right)+I_{Q_{BB^{\prime }}}\left(A;B^{\prime }|B,D,E\right)\right]\overset{\left(b\right)}{=}\\ \; & \min_{Q_{BB^{\prime }}\in \Delta_{P_{BB^{\prime }}}}\left[I_{Q_{B}}\left(A;B|D,E\right)+I_{Q_{BB^{\prime }}}\left(A;B^{\prime }|B,D,E\right)\right]. \end{array}$$

Equality (a) follows from the chain rule of mutual information. Equality (b) holds because $I_{Q_{BB^{\prime }}}\left(A;B|D,E\right)$ does not depend on $B^{\prime }$ and can be calculated with the marginal $Q_{B}$, marginalizing $Q_{BB^{\prime }}$ on $B^{\prime }$. Note that $Q_{B}\in \Delta_{P_{B}}$. Since $I_{P_{BB^{\prime }}}\left(A;B^{\prime }|B,E\right)$ is null, $P(A,B,B^{\prime },E)$ factorizes as $P\left(B^{\prime }|B,E\right)P\left(A,B,E\right)$. For any distribution ${\tilde{Q}}_{B}\in \Delta_{P_{B}}$, which preserves P(A,D,E) and P(A,B,E), a distribution can be constructed as ${\tilde{Q}}_{BB^{\prime }}\equiv P\left(B^{\prime }|B,E\right){\tilde{Q}}_{B}$, such that ${\tilde{Q}}_{BB^{\prime }}\in \Delta_{P_{BB^{\prime }}}$, since ${\tilde{Q}}_{BB^{\prime }}$ continues to preserve P(A,D,E) and $P(A,B,B^{\prime },E)$ is preserved by construction. Also by construction, $I_{{\tilde{Q}}_{BB^{\prime }}}\left(A;B^{\prime }|B,D,E\right)=0$ for any ${\tilde{Q}}_{BB^{\prime }}$ created from any ${\tilde{Q}}_{B}\in \Delta_{P_{B}}$. In particular, this holds for the distribution ${\tilde{Q}}_{BB^{\prime }}^{*}$ constructed from ${\tilde{Q}}_{B}^{*}$ that minimizes $I_{{\tilde{Q}}_{B}}\left(A;B|D,E\right)$, which determines I(A;B∖∖D|E). The distribution ${\tilde{Q}}_{BB^{\prime }}^{*}$ minimizes the first term in the r.h.s of Equation (4) and, given the non-negativity of mutual information, it also minimizes the second term, hence providing the minimum in $\Delta_{P_{BB^{\prime }}}$. Accordingly, $I(A;B,B^{\prime }\setminus \setminus D|E)$=I(A;B∖∖D|E). The monotonicity of unique information on the non-conditioning predictor (Lemma 2) leads to $I(A;B,B^{\prime }\setminus \setminus D|E)\geq I(A;B^{\prime }\setminus \setminus D|E)$. □

A related data processing inequality has already been previously derived for the unconditional unique information in the case of $I(A,D;B^{\prime }|B)=0$, with E=∅[39]. Differently, Proposition 2 formulates a data processing inequality for the case $I(A;B^{\prime }|B,E)=0$. When E=∅, Proposition 2 states a weaker requirement for the existence of an inequality, given the *decomposition axiom* of the mutual information [27]. As we will now see in Section 3.2, Proposition 2 will allow us to apply the unique information data processing inequality in cases in which $I(A;B^{\prime }|B,E)=0$. In particular, $I(A;B,B^{\prime }\setminus \setminus D|E)\geq I(A;B^{\prime }\setminus \setminus D|E)$ allows us to obtain a lower bound when B contains hidden variables that we want to eliminate in order to have a testable groups-decomposition inequality. In contrast, the application of the standard data processing inequality of the mutual information $I\left(A;B,B^{\prime }|D,E\right)\geq I\left(A;B^{\prime }|D,E\right)$ requires $I(A;B^{\prime }|B,D,E)=0$, and hence the two types of data processing inequalities may be applicable in different cases to eliminate B. This will be fully appreciated in Propositions 5 and 6. Note that this application of the unique information measure of Equation (2) to eliminate hidden variables is not restrained by the role of the measure in the mutual information decomposition and by considerations about which alternative decompositions optimally quantify the different components [30,35].

### 3.2. Inequalities Involving Sums of Information Terms from Groups

**Figure 2:**
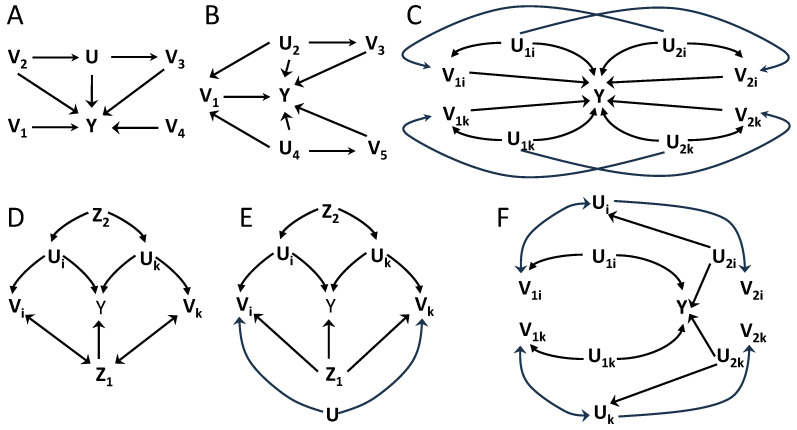
Examples of applications of Proposition 3 (**A**–**C**) and Proposition 4 (**D**–**F**) to obtain testable inequalities. The causal graphs allow verifying if the required conditional independence conditions are fulfilled by using d-separation. Variable *Y* is the target variable, observable variables are denoted by *V*, hidden variables by *U*, and conditioning variables by *Z*. For all examples, the composition of groups is described in the main text. For graphs using subindexes *i*, *k* to display two concrete groups, those are representative of the same causal structure for all groups that compose the system. In those graphs, variables with no subindex have the same connectivity with all groups. Bidirectional arrows indicate common hidden parents not included in any group.

**Figure 3:**
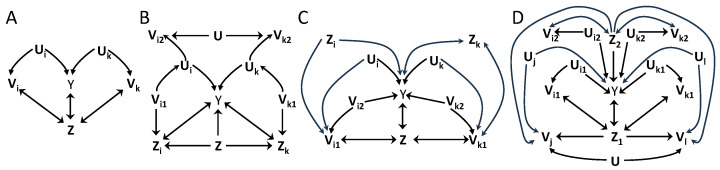
Examples of the application of Proposition 5 (**A**–**C**) and Proposition 6 (**D**) to obtain testable inequalities. Notation is analogous to Figure 2. The composition of groups is described in the main text.

In this section, we extend Proposition 1 in several ways. Propositions 3–6 present subsequent generalizations, all subsumed by Proposition 6. We present these generalizations progressively to better appreciate the new elements. For these Propositions, examples are displayed in Figure 2 and Figure 3 and explained in text after the enunciation of each Proposition. Which Proposition is illustrated by each example is indicated in the figure caption and in the main text. The objective of these generalizations is twofold: First, to derive new testable inequalities for causal structures not producing a testable inequality from Proposition 1. Second, to find inequalities with higher inferential power, even when some already exist. These objectives are achieved introducing inequalities with less constringent requirements of conditional independence and using data processing inequalities to substitute certain variables from $A_{\left[n\right]}$, so that the conditions of independence are fulfilled or the number of intersections is reduced and lower values in d are obtained. The first extension relaxes the conditions $A_{i}⊥A_{j}\setminus A_{i}|Z$∀i,j required in Proposition 1:

**Proposition 3.**  *(Weaker conditions of independence through group augmentation for a decomposition of information from groups with conditionally independent non-shared components): Consider a collection of groups $A_{\left[n\right]}$, a conditioning set Z, and target variables Y as in Proposition 1. Consider that for each group $A_{i}$ a group $B_{i}$ exists, such that $A_{i}\subseteq B_{i}$ and $B_{i}$ can be partitioned in two disjoint subsets $B_{i}=\left\{B_{i}^{\left(1\right)},B_{i}^{\left(2\right)}\right\}$ such that $B_{i}^{\left(1\right)}$ fulfills the conditions of independence $B_{i}^{\left(1\right)}⊥B_{j}^{\left(1\right)}\setminus B_{i}^{\left(1\right)}|Z$ and $B_{i}^{\left(2\right)}$ the conditions $B_{i}^{\left(2\right)}⊥B_{j}\setminus B_{i}^{\left(2\right)}|B_{i}^{\left(1\right)}Z$∀i,j, and such that $B_{\left[n\right]}^{\left(1\right)}\equiv \left\{B_{1}^{\left(1\right)},\cdots ,B_{n}^{\left(1\right)}\right\}$ and $B_{\left[n\right]}^{\left(2\right)}\equiv \left\{B_{1}^{\left(2\right)},\cdots ,B_{n}^{\left(2\right)}\right\}$ are disjoint. Define the maximal values $d_{B_{i}}$ like in Proposition 1 but for the augmented groups $B_{\left[n\right]}\equiv \left\{B_{1},\cdots ,B_{n}\right\}$. Then, the conditional information that $B_{\left[n\right]}$ has about the target variables Y given Z is bounded from below by:*

$$I\left(Y;B_{\left[n\right]}|Z\right)\geq \sum_{i=1}^{n}\frac{1}{d_{B_{i}}}I\left(Y;B_{i}|Z\right)\geq \sum_{i=1}^{n}\frac{1}{d_{B_{i}}}I\left(Y;A_{i}|Z\right).$$

**Proof.**  The proof is provided in Appendix A. □

The contribution of Proposition 3 is to relax the conditional independence requirements $A_{i}⊥A_{j}\setminus A_{i}|Z$. Analogous conditions remain for $B_{i}^{\left(1\right)}$, but $B_{i}^{\left(2\right)}$ needs to fulfill the conditions $B_{i}^{\left(2\right)}⊥B_{j}\setminus B_{i}^{\left(2\right)}|B_{i}^{\left(1\right)}Z$∀i,j. This means that the variables in $B_{i}^{\left(1\right)}$ are used to separate the variables in $B_{i}^{\left(2\right)}$ from other groups. If $B_{i}^{\left(2\right)}$ is empty for all *i*, Proposition 3 reduces to Proposition 1.

Another difference between Propositions 1 and 3 regards the role of hidden variables. Assume that each $A_{i}$ is formed by $\left\{V_{i},U_{i}\right\}$, where $U_{i}$ are hidden variables and $V_{i}$ observable variables. In Proposition 1, the requirement that the variables are observable is not fundamental and could be removed. However, to obtain a testable inequality, monotonicity of mutual information would need to be applied to reduce each term $I(Y;A_{i}|Z)$ to its estimable lower bound $I(Y;V_{i}|Z)$ that does not contain the hidden variables $U_{i}$. On the other hand, the fulfillment of $A_{i}⊥A_{j}\setminus A_{i}|Z$ implies $V_{i}⊥V_{j}\setminus V_{i}|Z$, and reducing $A_{i}$ to $V_{i}$ can only decrease the number of intersections, and hence $d_{V_{\left[n\right]}}$ values are equal or smaller than $d_{A_{\left[n\right]}}$. Therefore, with Proposition 1, there is no advantage in including hidden variables. When testing Proposition 1 for a hypothesis of the underlying causal structure (and related independencies), it is equally or more powerful to use $V_{\left[n\right]}$ than $A_{\left[n\right]}$.

This changes in Proposition 3, since $B_{i}^{\left(1\right)}$ appears in the conditioning side of the independencies that constrain $B_{i}^{\left(2\right)}$. If hidden variables within $B_{i}^{\left(1\right)}$ are necessary to create the independencies for $B_{i}^{\left(2\right)}$, it is not possible to reduce each group to its subset of observable variables. Note that, for a hypothesized causal structure, whether the independence conditions required by Proposition 3 are fulfilled can be verified without observing the hidden variables by using the d-separation criterion on the causal graph, assuming d-separation implies independence. The actual estimation of mutual information values is only needed when testing an inequality from the data.

If $B_{\left[n\right]}$ includes hidden variables, in general $I(Y;B_{\left[n\right]}|Z)$ cannot be estimated and H(Y|Z) is used as an upper bound. For the r.h.s. of the inequality, a lower bound is obtained by monotonicity of the mutual information, removing the hidden variables. In general, a testable inequality has the form

(5) $$H\left(Y|Z\right)\geq \sum_{i=1}^{n}\frac{1}{d_{B_{i}}}I\left(Y;V_{i}|Z\right),$$

with $V_{i}\subseteq B_{i}$ being the observable variables within each group. In the case that $I(Y;B_{\left[n\right]}|Z)$$=I(Y;V_{\left[n\right]}|Z)$, that is, if the hidden variables do not add information, then a testable tighter upper bound is available using $I(Y;V_{\left[n\right]}|Z)$. Importantly, the values $d_{B_{\left[n\right]}}$ are determined using the groups in $B_{\left[n\right]}$. Since $A_{i}\subseteq B_{i}$, group augmentation comes at the price that $d_{B_{\left[n\right]}}$ are equal or higher than $d_{A_{\left[n\right]}}$, but the conditional independence requirements may not be fulfilled without it. Note also that the partition $B_{i}=\left\{B_{i}^{\left(1\right)},B_{i}^{\left(2\right)}\right\}$ is not known a priori, but determined in the process of finding suitable augmented groups that fulfill the conditions.

We examine some examples before further generalizations. Throughout all figures, we will read independencies from the causal structures using d-separation, assuming faithfulness. In Figure 2A, consider groups $A_{1}=\left\{V_{1},V_{2}\right\}$ and $A_{2}=\left\{V_{3},V_{4}\right\}$, and Z=∅. Proposition 1 is not applicable due to $V_{2}$ 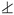
$V_{3}$. Augmenting the groups to $B_{1}^{\left(1\right)}=B_{2}^{\left(1\right)}=\left\{U\right\}$, $B_{1}^{\left(2\right)}=\left\{V_{1},V_{2}\right\}$, and $B_{2}^{\left(2\right)}=\left\{V_{3},V_{4}\right\}$ the conditions of Proposition 3 are fulfilled, as can be verified by d-separation. Coefficients are determined by d={2,2} due to the intersection of the groups in *U*. Note that hidden variables are not restricted to be hidden common ancestors, and here *U* is a mediator between $V_{2}$ and $V_{3}$. In Figure 2B, consider groups $A_{1}=\left\{V_{1}\right\}$, $A_{2}=\left\{V_{3}\right\}$, $A_{3}=\left\{V_{5}\right\}$, which do not fulfill the conditions of Proposition 1. Augmenting the groups to $B_{1}^{\left(1\right)}=\left\{U_{2},U_{4}\right\}$, $B_{1}^{\left(2\right)}=\left\{V_{1}\right\}$, $B_{2}^{\left(1\right)}=\left\{U_{2}\right\}$, $B_{2}^{\left(2\right)}=\left\{V_{3}\right\}$, $B_{3}^{\left(1\right)}=\left\{U_{4}\right\}$, and $B_{3}^{\left(2\right)}=\left\{V_{5}\right\}$ the conditions are fulfilled. Maximal intersection values are d={2,2,2}. In both examples the upper bound is H(Y) since $I(Y;B_{\left[n\right]})$ cannot be estimated due to hidden variables.

We also consider scenarios with more groups. Figure 2C represents 2N groups organized in pairs, with subindexes i,k indicating two particular pairs. The 2N groups are defined in pairs, with $A_{1j}=\left\{V_{1j}\right\}$ and $A_{2j}=\left\{V_{2j}\right\}$, j=1,⋯,N. The causal structure is the same across pairs, but the mechanisms generating the variables beyond the causal structure can possibly differ. Proposition 1 is not fulfilled since $V_{1j}$
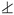
$V_{2j}$. Groups can be augmented to $B_{j^{\prime }j}^{\left(1\right)}=\left\{U_{1j},U_{2j}\right\}$, $B_{j^{\prime }j}^{\left(2\right)}=\left\{V_{j^{\prime }j}\right\}$, for $j^{\prime }=1,2$. Proposition 3 then holds with d=2 for all 2N groups. The pairs of groups contribute to the sum as $1/2[I\left(Y;V_{1j},U_{1j},U_{2j}\right)+I\left(Y;V_{2j},U_{1j},U_{2j}\right)]$, which in the testable inequality of the form of Equation (5) reduces to $1/2[I\left(Y;V_{1j}\right)+I\left(Y;V_{2j}\right)]$. The upper bound to the sum of 2N terms is H(Y). This inequality provides causal inference power because $V_{1j}⊥V_{2j}|U_{1j},U_{2j}$ for all *j* is not directly testable. As previously indicated, the inference power of an inequality emanates from the possibility to discard causal structures that do not fulfill it. Note that for this system an alternative is to define *N* groups instead of 2N groups, each as $A_{j}^{\prime }=\left\{V_{1j},V_{2j}\right\}$. In this case Proposition 1 is already applicable with the coefficients being all 1, since $V_{1i},V_{2i}⊥V_{1j},V_{2j}$ for all i≠j. For this inequality, each of the N groups contributes with $I\left(Y;V_{1j}\right)+I\left(Y;V_{2j}|V_{1j}\right)$, and since there are no hidden variables the l.h.s. is $I(Y;A_{\left[n\right]}^{\prime })$. However, this latter inequality holds for any causal structure that fulfills $V_{1i},V_{2i}⊥V_{1j},V_{2j}$ for all i≠j. Given that these independencies do not involve hidden variables, they are directly testable from data, so that the latter inequality does not provide additional inference power, in contrast to the former one.

We now continue with further generalizations. Group augmentation in Proposition 3 cannot decrease the values of the maximal number of intersections. We now describe how the data processing inequalities in Lemma 1(i) and Proposition 2 can be used to substitute variables within the groups, potentially reducing the number of intersections. We start with the data processing inequality for the conditional mutual information.

**Proposition 4.**  *(Decomposition of information from groups modified with the conditional mutual information data processing inequality): Consider a collection of groups $A_{\left[n\right]}$, a conditioning set Z, and target variables Y as in Proposition 1. Consider that for some group $A_{i}$ a group $B_{i}$ exists such that $Y⊥A_{i}\setminus B_{i}|B_{i}Z$, with $A_{i}\setminus B_{i}\neq \emptyset$. Define $B_{\left[n\right]}$ as the collection of groups that replaces $A_{i}$ by $B_{i}$ for those following the previous independence condition. If $B_{\left[n\right]}$ fulfills the conditions of Proposition 3, the inequality derived for $B_{\left[n\right]}$ also provides an upper bound for the sum of the information provided by the groups in $A_{\left[n\right]}$:*

$$H\left(Y|Z\right)\geq I\left(Y;B_{\left[n\right]}|Z\right)\geq \sum_{i=1}^{n}\frac{1}{d_{B_{i}}}I\left(Y;B_{i}|Z\right)\geq \sum_{i=1}^{n}\frac{1}{d_{B_{i}}}I\left(Y;A_{i}|Z\right).$$

**Proof.**  The proof applies Proposition 3 to $B_{\left[n\right]}$ followed by the data processing inequality of Lemma 1(i) to each term within the sum in which $A_{i}$ and $B_{i}$ are different. Given that $Y⊥A_{i}\setminus B_{i}|B_{i}Z$ implies $I\left(Y;B_{i}|Z\right)\geq I\left(Y;A_{i}|Z\right)$, their sum is also smaller or equal. □

Proposition 3 envisaged cases in which the conditions of independence of Proposition 1 were not fulfilled for a collection $A_{\left[n\right]}$ and augmentation allowed fulfilling weaker conditions, even if with higher $d_{B_{\left[n\right]}}$ values compared to $d_{A_{\left[n\right]}}$. Proposition 4 is useful not only when the conditions of independence are not fulfilled for $A_{\left[n\right]}$, but more generally if some values in $d_{B_{\left[n\right]}}$ are lower than in $d_{A_{\left[n\right]}}$, hence providing a tighter inequality. Including hidden variables in $B_{\left[n\right]}$ is beneficiary when replacing observed by hidden variables leads to fewer intersections. The procedures of Proposition 3 and 4 can be combined, that is, starting with $A_{\left[n\right]}$ that contains only observable variables, a new collection can be constructed adding new variables and removing others from $A_{\left[n\right]}$, ending with $B_{\left[n\right]}$ that contains both observable and hidden variables. The collection $B_{\left[n\right]}$ fulfilling the conditions of Proposition 3 may even contain only hidden variables, and a testable inequality is obtained as long as the data processing inequality allows calculating observable lower bounds for all terms in the sum.

Figure 2D–F are examples of Proposition 4. Again we consider cases with *N* groups with equal causal structure and use indexes i,k to represent two concrete groups. In Figure 2D, with $A_{j}=\left\{V_{j}\right\}$, Proposition 3 does not apply for $A_{\left[n\right]}$ conditioning on $\left\{Z_{1},Z_{2}\right\}$ because $V_{i}$
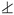
$V_{j}|Z_{1},Z_{2}$, for all i,j. However, given that $Y⊥V_{j}|U_{j},Z_{1},Z_{2}$, each $V_{j}$ can be replaced to build $B_{j}=\left\{U_{j}\right\}$, and since $U_{i}⊥U_{j}|Z_{1},Z_{2}$, for all i,j Proposition 3 applies after using Proposition 4 to create $B_{\left[n\right]}$. A testable inequality is derived with upper bound $H(Y|Z_{1},Z_{2})$ and a sum of terms $I(Y;V_{j}|Z_{1},Z_{2})$, each being a lower bound of $I(Y;U_{j}|Z_{1},Z_{2})$ given the data processing inequality that follows from $Y⊥V_{j}|U_{j},Z_{1},Z_{2}$. The coefficients are $d_{B_{\left[n\right]}}=1$. Therefore, in this case Proposition 4 results in an inequality when no inequality held for $A_{\left[n\right]}$. In Figure 2E, the same procedure relies on $Y⊥V_{j}|U_{j},Z_{1},Z_{2}$ and $U_{i}⊥U_{j}|Z_{1},Z_{2}$ to use $B_{j}=\left\{U_{j}\right\}$ to create a testable inequality with l.h.s. $H(Y|Z_{1},Z_{2})$ and the sum of terms $I(Y;V_{j}|Z_{1},Z_{2})$ in the r.h.s. with $d_{B_{\left[n\right]}}=1$. Note that by *U*, which has no subindex, we represent in Figure 2E a hidden common driver of all *N* groups, not only the displayed i,k. In this example Proposition 3 could have been directly applied without using Proposition 4 if augmenting $A_{j}=\left\{V_{j}\right\}$ to $B_{j}^{\prime }=\left\{V_{j},U\right\}$, with $B_{j}^{\prime (1)}=\left\{U\right\}$ and $B_{j}^{\prime (2)}=\left\{V_{j}\right\}$, since $V_{i}⊥V_{j}|U,Z_{1},Z_{2}$. However, $d_{B_{\left[n\right]}^{\prime }}=N$, since all groups $B_{j}^{\prime }$ intersect in *U*. Therefore, in this case an inequality already exists without applying Proposition 4, but its use allows replacing $d_{B_{\left[n\right]}^{\prime }}=N$ by $d_{B_{\left[n\right]}}=1$, hence creating a tighter inequality with higher causal inference power.

In Figure 2F, again we consider 2N groups, consisting of *N* pairs with the same causal structure across pairs and indices i,k representing two of these pairs. For groups $A_{j^{\prime }j}=\left\{V_{j^{\prime }j}\right\}$, with $j^{\prime }=1,2$ and j=1,...,N, Proposition 3 is directly applicable for $B_{j^{\prime }j}^{\left(1\right)}=\left\{U_{j}\right\}$ and $B_{j^{\prime }j}^{\left(2\right)}=\left\{V_{j^{\prime }j}\right\}$, with $d_{B_{\left[n\right]}}=2$. The data processing inequalities associated with $Y⊥V_{j^{\prime }j}|U_{j^{\prime }j}$ allow applying Proposition 4 to obtain an inequality for the groups $B_{j^{\prime }j}^{\prime }=B_{j^{\prime }j}^{\prime \left(1\right)}=\left\{U_{j^{\prime }j}\right\}$, which $d_{B_{\left[n\right]}^{\prime }}=1$.

Proposition 4 relies on the data processing inequality of the conditional mutual information. The data processing inequality of unique information can also be used for the same purpose, and both data processing inequalities can be combined applying them to different groups.

**Proposition 5.**  *(Decomposition of information from groups modified using across different groups the conditional or unique information data processing inequality): Consider a collection of groups $A_{\left[n\right]}$, a conditioning set Z, and target variables Y as in Proposition 1. Consider a subset of groups such that for $A_{i}$ a group $B_{i}$ exists such that, for some $Z_{i}^{\left(1\right)}\subseteq Z$, $Y⊥A_{i}\setminus B_{i}|B_{i}Z_{i}^{\left(1\right)}$, with $A_{i}\setminus B_{i}\neq \emptyset$. Define $B_{\left[n\right]}$ as the collection of groups that replaces $A_{i}$ by $B_{i}$ for those following the previous independence conditions. Define $Z_{i}^{\left(1\right)}\equiv Z$ for the unaltered groups and $Z_{i}^{\left(2\right)}\equiv Z\setminus Z_{i}^{\left(1\right)}$ for all groups. If $B_{\left[n\right]}$ fulfills the conditions of Proposition 3, the inequality derived for $B_{\left[n\right]}$ also provides an upper bound for a sum combining conditional and unique information terms for different groups in $A_{\left[n\right]}$:*

$$\begin{array}{cc} \; & H\left(Y|Z\right)\geq I\left(Y;B_{\left[n\right]}|Z\right)\geq \sum_{i=1}^{n}\frac{1}{d_{B_{i}}}I\left(Y;B_{i}|Z\right)\geq \\ \; & \sum_{\left\{i:|Z_{i}^{\left(2\right)}|=0\right\}}\frac{1}{d_{B_{i}}}I\left(Y;A_{i}|Z\right)+\sum_{\left\{i:|Z_{i}^{\left(2\right)}|>0\right\}}\frac{1}{d_{B_{i}}}I(Y;A_{i}\setminus \setminus Z_{i}^{\left(2\right)}\left|Z_{i}^{\left(1\right)}\right). \end{array}$$

**Proof.**  The proof applies Proposition 3 to $B_{\left[n\right]}$ and then both types of data processing inequalities depending on which one holds for different groups:

(6) $$\begin{array}{cc} \; & \sum_{i=1}^{n}\frac{1}{d_{B_{i}}}I\left(Y;B_{i}|Z\right)\overset{\left(a\right)}{\geq }\sum_{\left\{i:|Z_{i}^{\left(2\right)}|=0\right\}}\frac{1}{d_{B_{i}}}I\left(Y;B_{i}|Z\right)+\sum_{\left\{i:|Z_{i}^{\left(2\right)}|>0\right\}}\frac{1}{d_{B_{i}}}I(Y;B_{i}\setminus \setminus Z_{i}^{\left(2\right)}\left|Z_{i}^{\left(1\right)}\right)\overset{\left(b\right)}{\geq }\\ \; & \sum_{\left\{i:|Z_{i}^{\left(2\right)}|=0\right\}}\frac{1}{d_{B_{i}}}I\left(Y;A_{i}|Z\right)+\sum_{\left\{i:|Z_{i}^{\left(2\right)}|>0\right\}}\frac{1}{d_{B_{i}}}I(Y;A_{i}\setminus \setminus Z_{i}^{\left(2\right)}\left|Z_{i}^{\left(1\right)}\right). \end{array}$$

Inequality (a) follows from the unique information always being equal to or smaller than the conditional mutual information (Equation (3)). Inequality (b) applies the conditional mutual information data processing inequality to those groups with $A_{i}$ different than $B_{i}$ but $|Z_{i}^{\left(2\right)}|=0$, and the unique information data processing inequality to those groups with $|Z_{i}^{\left(2\right)}|>0$. □

Proposition 5 is useful when the conditions of independence required to apply Proposition 3 do not hold for $A_{\left[n\right]}$. It can also be useful to obtain inequalities with higher causal inferential power if $d_{B_{\left[n\right]}}$ are smaller than $d_{A_{\left[n\right]}}$, even if Proposition 3 is directly applicable. By definition, the terms $I(Y;A_{i}\setminus \setminus Z_{i}^{\left(2\right)}|Z_{i}^{\left(1\right)})$ are equal to or smaller than $I(Y;A_{i}|Z)$, which can only decrease the lower bound, but the data processing inequality may hold only for the unique information and not the conditional information term. Note that the partition $\left\{Z_{i}^{\left(1\right)},Z_{i}^{\left(2\right)}\right\}$ can be group-specific and selected such that data processing inequalities can be applied.

Figure 3A shows an example of the application of the data processing inequality of unique information. For $A_{j}=\left\{V_{j}\right\}$, Proposition 3 does not apply to $I(Y;A_{\left[n\right]}|Z)$ because $V_{i}$
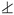
$V_{k}|Z$. The data processing inequality of conditional mutual information does not hold with Y
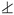
$V_{i}|U_{i}Z$. This data processing inequality could be used adding to $U_{i}$ the latent common parent in Y↔Z, but this variable would be shared by all augmented groups $B_{i}$, leading to an intersection of all *N* groups. Alternatively, the data processing inequality holds for the unique information with $I(Y;U_{j}\setminus \setminus Z)\geq I(Y;V_{j}\setminus \setminus Z)$, and $U_{i}⊥U_{j}|Z$ for all i≠j. Proposition 5 is applied with $Z_{j}^{\left(1\right)}=\emptyset$, $Z_{j}^{\left(2\right)}=\left\{Z\right\}$, and $B_{j}=B_{j}^{\left(1\right)}=\left\{U_{j}\right\}$, ∀j. This leads to an inequality with H(Y|Z) as upper bound and the sum of terms $I(Y;V_{j}\setminus \setminus Z)$ at the r.h.s. with coefficients determined by $d_{B_{\left[n\right]}}=1$. In Figure 3B, taking $A_{j}=\left\{V_{j1},V_{j2}\right\}$∀j and defining the conditioning set $Z=\{Z,Z_{1},...,Z_{N}\}$, we have $V_{i2}$
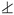
$V_{k2}|Z$ and $V_{j1},V_{j2}$
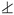
$Y|U_{j}Z$. On the other hand, $V_{j1},V_{j2}⊥Y|U_{j}Z\setminus Z_{j}$, so that the data processing can be applied with the unique information and Proposition 5 is applied with $Z_{j}^{\left(1\right)}=Z\setminus Z_{j}$, $Z_{j}^{\left(2\right)}=\left\{Z_{j}\right\}$ and $B_{j}=B_{j}^{\left(1\right)}=\left\{U_{j}\right\}$. An inequality exists given that $U_{i}⊥U_{k}|Z$, and the testable inequality has an upper bound H(Y|Z) and at the r.h.s. the sum of terms $I(Y;V_{j1}V_{j2}\setminus \setminus Z_{j}|Z\setminus Z_{j})$, with $d_{B_{\left[n\right]}}=1$.

In Figure 3C, we examine an example in which groups differ in the causal structure of the conditioning variable $Z_{j}$: For the groups of the type of group *i*, $Z_{i}$ is a common parent of *Y* and $V_{i1}$. For the groups of the type of *k*, $Z_{k}$ is a collider in a path between *Y* and $V_{k1}$. Consider *M* groups of the former type and N−M of the latter. We examine the existence of an inequality for groups defined as $A_{j}=\left\{V_{j1},V_{j2}\right\}$∀j, with $Z=\{Z,Z_{1},\cdots ,Z_{N}\}$. Proposition 3 cannot be applied to $I(Y;A_{\left[n\right]}|Z)$ because $V_{i1}$
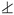
$V_{j1}|Z$ for all i≠j. The mutual information data processing inequality is not applicable to substitute $V_{j1}$ because Y
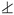
$V_{j1}|U_{j}V_{j2}Z$. However, for the *M* groups like *i*, the independence $Y⊥V_{j1}|U_{j}V_{j2}Z\setminus Z$ leads to the data processing inequality $I(Y;U_{j}V_{j2}\setminus \setminus Z|Z\setminus Z)$$\geq I(Y;V_{j1}V_{j2}\setminus \setminus Z|Z\setminus Z)$. For these groups, $Z_{j}^{\left(1\right)}=Z\setminus Z$ and $Z_{j}^{\left(2\right)}=\left\{Z\right\}$. For the N−M groups like *k*, the independence $Y⊥V_{j1}|U_{j}V_{j2}Z\setminus \left\{Z,Z_{j}\right\}$ leads to $I(Y;U_{j}V_{j2}\setminus \setminus Z,Z_{j}|Z\setminus \left\{Z,Z_{j}\right\})$$\geq I(Y;V_{j1}V_{j2}\setminus \setminus Z,Z_{j}|Z\setminus \left\{Z,Z_{j}\right\})$. For these groups $Z_{j}^{\left(1\right)}=Z\setminus \left\{Z,Z_{j}\right\}$ and $Z_{j}^{\left(2\right)}=\left\{Z,Z_{j}\right\}$. In all cases the modified groups are $B_{j}=B_{j}^{\left(1\right)}=\left\{U_{j},V_{j2}\right\}$, which fulfill the requirement $U_{j},V_{j2}⊥U_{i},V_{i2}|Z$ for all i≠j needed to apply Proposition 3. The testable inequality that follows from Proposition 5 has upper bound H(Y|Z) and in the sum at the r.h.s. has *M* terms of the form $I(Y;V_{j1}V_{j2}\setminus \setminus Z|Z\setminus Z)$ and N−M terms of the form $I(Y;V_{j1}V_{j2}\setminus \setminus Z,Z_{j}|Z\setminus \left\{Z,Z_{j}\right\})$. The coefficients are determined by $d_{B_{\left[n\right]}}=1$.

Proposition 5 combines both types of data processing inequalities, but only across different groups. Our last extension of Proposition 1 combines both types across and within groups. For each group, we introduce a disjoint partition into $m_{i}$ subgroups $A_{i}=\left\{A_{i}^{\left(1\right)},\cdots ,A_{i}^{\left(m_{i}\right)}\right\}$ and define $A_{i}^{\left(0\right)}\equiv \emptyset$. Subgroups are analogously defined for $Z_{i}$, also with $Z_{i}^{\left(0\right)}\equiv \emptyset$. In general, for any ordered set of vectors we use $V_{i}^{\left[k\right]}\equiv \left\{V_{i}^{\left(0\right)},V_{i}^{\left(1\right)},\cdots ,V_{i}^{\left(k\right)}\right\}$ to refer to all elements up to *k*, where in general $V_{i}^{\left(0\right)}$ can be nonempty.

**Proposition 6.**  *(Decomposition of information from groups modified with the conditional or unique information data processing inequality across and within groups): Consider a collection of groups $A_{\left[n\right]}$, a conditioning set Z, and a target variable Y as in Proposition 1. Consider that for each group $A_{i}$ there are disjoint partitions $A_{i}=\left\{A_{i}^{\left(1\right)},\cdots ,A_{i}^{\left(m_{i}\right)}\right\}$ and $Z=\{Z_{i}^{\left(1\right)},\cdots ,Z_{i}^{\left(m_{i}\right)}\}$, and a collection of sets of additional variables $C_{i}=\left\{C_{i}^{\left(0\right)},C_{i}^{\left(1\right)},...,C_{i}^{\left(m_{i}-1\right)}\right\}$, such that $Y⊥A_{i}^{\left(k\right)}|C_{i}^{\left[k\right]}Z_{i}^{\left[k\right]}A_{i}\setminus A_{i}^{\left[k\right]}$ for $k=1,\cdots ,m_{i}-1$. Define the collection $B_{\left[n\right]}$ with the modified groups $B_{i}=\left\{C_{i},A_{i}^{\left(m_{i}\right)}\right\}$. If $B_{\left[n\right]}$ fulfills the conditions of Proposition 3, the inequality derived for $B_{\left[n\right]}$ also provides an upper bound for sums combining conditional and unique information terms for different groups in $A_{\left[n\right]}$:*

$$\begin{array}{cc} \; & H\left(Y|Z\right)\geq I\left(Y;B_{\left[n\right]}|Z\right)\geq \sum_{i=1}^{n}\frac{1}{d_{B_{i}}}I\left(Y;B_{i}|Z\right)\geq \\ \; & \sum_{i=1}^{n}\frac{1}{d_{B_{i}}}I(Y;C_{i}^{\left[k_{i}\right]}A_{i}\setminus A_{i}^{\left[k_{i}\right]}\setminus \setminus Z\setminus Z_{i}^{\left[k_{i}\right]}|Z_{i}^{\left[k_{i}\right]})\geq \sum_{i=1}^{n}\frac{1}{d_{B_{i}}}I(Y;A_{i}\setminus \setminus Z\setminus Z_{i}^{\left(1\right)}\left|Z_{i}^{\left(1\right)}\right), \end{array}$$

*for $k_{i}\in \left\{1,\cdots ,m_{i}-1\right\}$.*

**Proof.**  The proof is provided in Appendix A. □

If $m_{i}=1$ for all *i*, then $A_{i}^{\left(1\right)}=A_{i}$, $Z_{i}^{\left(1\right)}=Z$, $B_{i}=\left\{C_{i}^{\left(0\right)},A_{i}\right\}$, and Proposition 6 reduces to Proposition 3. If $m_{i}=2$ and $Z_{i}^{\left(1\right)}=Z$ for all *i*, we recover Proposition 4, with $B_{i}=\left\{C_{i},A_{i}^{\left(2\right)}\right\}$. If $m_{i}=2$ for all *i* and $Z_{i}^{\left(1\right)}\subset Z$ for some *i*, we recover Proposition 5, with $B_{i}=\left\{C_{i},A_{i}^{\left(2\right)}\right\}$ and $Z_{i}^{\left(2\right)}=Z\setminus Z_{i}^{\left(1\right)}$. Like for previous propositions, some groups may be unmodified such that $B_{i}=A_{i}$.

The tightest inequality results from maximizing across $k_{i}\in \left\{1,\cdots ,m_{i}-1\right\}$ each term in the sum. In the proof of Proposition 6 in Appendix A we show that, when increasing $k_{i}\in \left\{1,\cdots ,m_{i}-1\right\}$, the terms $I(Y;C_{i}^{\left[k\right]}A_{i}\setminus A_{i}^{\left[k\right]}\setminus \setminus Z\setminus Z_{i}^{\left[k\right]}|Z_{i}^{\left[k\right]})$ are monotonically increasing. However, in general $C_{i}$ can contain hidden variables, which means that, to obtain a testable inequality, for each $k_{i}\in \left\{1,\cdots ,m_{i}-1\right\}$ each term needs to be substituted by its lower bound that quantifies the information in the subset of observable variables. For each group, the optimal $k_{i}$ leading to the tightest inequality will depend on the subset of observable variables $V_{i}^{\left(k_{i}\right)}\subseteq \left\{C_{i}^{\left[k_{i}\right]},A_{i}\setminus A_{i}^{\left[k_{i}\right]}\right\}$ and the corresponding values of $I(Y;V_{i}^{\left(k_{i}\right)}\setminus \setminus Z\setminus Z_{i}^{\left[k_{i}\right]}|Z_{i}^{\left[k_{i}\right]})$.

Figure 3D shows an example of application of Proposition 6. Like in Figure 3C, there are two types of groups with different causal structure. *M* groups have the structure of the variables with indexes i,k, and $A_{j^{\prime }}=\left\{V_{j^{\prime }1},V_{j^{\prime }2}\right\}$. The other N−M groups have the structure of the variables with indexes l,j, and $A_{j^{\prime }}=\left\{V_{j^{\prime }}\right\}$. The conditioning set selected is $Z=\{Z_{1},Z_{2}\}$. Proposition 3 cannot be applied directly because $V_{i1}$
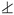
$V_{k1}|Z$ for all i≠k within the *M* groups, and $V_{j}$
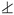
$V_{l}|Z$ for all j≠l within the N−M groups. Proposition 6 applies as follows. For the N−M groups, $m_{j^{\prime }}=2$ with $A_{j^{\prime }}^{\left(1\right)}=\left\{V_{j^{\prime }}\right\}$, $A_{j^{\prime }}^{\left(2\right)}=\emptyset$, $Z_{j^{\prime }}^{\left(1\right)}=Z$, and $B_{j^{\prime }}=C_{j^{\prime }}^{\left(1\right)}=\left\{U_{j^{\prime }}\right\}$. The independencies $Y⊥A_{i}^{\left(k\right)}|C_{i}^{\left[k\right]}Z_{i}^{\left[k\right]}A_{i}\setminus A_{i}^{\left[k\right]}$ for $k=1,\cdots ,m_{i}-1$ correspond in this case to $Y⊥V_{j^{\prime }}|ZU_{j^{\prime }}$, for k=1. For the other *M* groups, $m_{j^{\prime }}=3$ with $A_{j^{\prime }}^{\left(1\right)}=\left\{V_{j^{\prime }1}\right\}$, $A_{j^{\prime }}^{\left(2\right)}=\left\{V_{j^{\prime }2}\right\}$, $A_{j^{\prime }}^{\left(3\right)}=\emptyset$, $Z_{j^{\prime }}^{\left(1\right)}=\left\{Z_{2}\right\}$, $Z_{j^{\prime }}^{\left(2\right)}=\left\{Z_{1}\right\}$, $C_{j^{\prime }}^{\left(1\right)}=\left\{U_{j^{\prime }1}\right\}$, $C_{j^{\prime }}^{\left(2\right)}=\left\{U_{j^{\prime }2}\right\}$, and $B_{j^{\prime }}=\left\{U_{j^{\prime }1},U_{j^{\prime }2}\right\}$. The independencies involved are $Y⊥V_{j^{\prime }1}|Z_{2},U_{j^{\prime }1},V_{j^{\prime }2}$, for k=1, and $Y⊥V_{j^{\prime }2}|Z,U_{j^{\prime }1},U_{j^{\prime }2}$, for k=2.

Proposition 6 applies because with $B_{\left[n\right]}$ defined as $B_{j^{\prime }}=\left\{U_{j^{\prime }}\right\}$ for the N−M groups and $B_{j^{\prime }}=\left\{U_{j^{\prime }1},U_{j^{\prime }2}\right\}$ for the *M* groups, the requirements of independence of Proposition 3 are fulfilled, in particular $B_{i}⊥B_{j}|Z$ for all i≠j. The terms $I(Y;B_{j^{\prime }}|Z)$ for the N−M groups are $I(Y;U_{j^{\prime }}|Z_{1},Z_{2})$ and are substituted by lower bounds $I(Y;V_{j^{\prime }}|Z_{1},Z_{2})$ in the testable inequality. For the *M* groups, we have the subsequent sequence of inequalities: $I(Y;U_{j^{\prime }1},U_{j^{\prime }2}|Z_{1},Z_{2})$≥$I(Y;U_{j^{\prime }1},V_{j^{\prime }2}|Z_{1},Z_{2})$≥$I(Y;U_{j^{\prime }1},V_{j^{\prime }2}\setminus \setminus Z_{1}|Z_{2})$≥$I(Y;V_{j^{\prime }1},V_{j^{\prime }2}\setminus \setminus Z_{1}|Z_{2})$. The first inequality follows from the independence for k=2, the second from the unique information being equal or smaller than the conditional information, and the third from the independence for k=1. Considering that a testable inequality can only contain observable variables, for the *M* groups the terms in the sum can be $I(Y;V_{j^{\prime }1},V_{j^{\prime }2}\setminus \setminus Z_{1}|Z_{2})$ or $I(Y;V_{j^{\prime }2}|Z_{1},Z_{2})$, depending on which one is higher. The coefficients are determined by $d_{B_{\left[n\right]}}=1$ and the resulting testable inequality has upper bound $H(Y|Z_{1},Z_{2})$.

Overall, Propositions 4–6 further extend the cases in which groups-decomposition inequalities of the type of Proposition 1 can be derived. Our Proposition 1 extends Proposition 1 of [27] to allow conditioning sets, Proposition 3 further weakens the conditions of independence required in Proposition 1, and Propositions 4–6 use data processing inequalities to obtain testable inequalities from groups-decompositions derived comprising hidden variables, which can be more powerful than inequalities directly derived without comprising hidden variables. In Figure 2 and Figure 3, we have provided examples of causal structures for which these new groups-decompositions inequalities exist. In all these cases, the use of our groups-decomposition inequalities increases the set of available inequality tests that can be used to reject hypothesized causal structures underlying data.

### 3.3. Inequalities Involving Sums of Information Terms from Ancestral Sets

We now examine inequalities involving ancestral sets as in Theorem 1 of Steudel and Ay [27], which we reviewed in our Theorem 1 (Section 2.4). We extend this theorem allowing for a conditioning set Z and adding flexibility on how ancestral sets are constructed, as well as allowing the selection of reduced ancestral sets that exclude some variables. Like for Theorem 1, we will use $an_{G}\left(A_{\left[n\right]}\right)\equiv \left\{an_{G}\left(A_{1}\right),\cdots ,an_{G}\left(A_{n}\right)\right\}$ to indicate the collection of all ancestral sets in graph *G* from the collection of groups $A_{\left[n\right]}\equiv \left\{A_{1},\cdots ,A_{n}\right\}$.

The extension of Theorem 1 to allow for a conditioning set Z requires an extension of the notion of ancestral set that will be used to determine the coefficients in the inequalities. The intuition for this extension is that conditioning on Z can introduce new dependencies between groups, in particular when a variable $Z_{j}\in Z$ is a common descendant of several ancestral groups, and hence conditioning on it activates paths in which it is a collider. The coefficients need to take into account that common information contributions across ancestral groups can originate from these new dependencies. At the same time, conditioning can also block paths that created dependencies between the ancestral groups. To also account for this, we will not only consider ancestral sets in the original graph *G*, but in any graph $G^{\prime }=G_{\underline{Z^{\prime }}}$, with $Z^{\prime }\subseteq Z$. The graph $G_{\underline{Z^{\prime }}}$ is constructed by removing from *G* all the outgoing arrows from nodes in $Z^{\prime }$. This has an effect equivalent to conditioning on $Z^{\prime }$ with regard to eliminating dependencies enabled by paths through $Z^{\prime }$ in which the variables in $Z^{\prime }$ are noncolliders, since removing those arrows deactivates the paths. To account for these effects of conditioning on Z, for each $Z_{j}\in Z$ we define an augmented ancestral set of the groups $A_{i}\in A_{\left[n\right]}$ as follows:

(7) $$an_{G^{\prime }}\left(A_{i};Z_{j}\right)\equiv \left\{\begin{array}{c} an_{G^{\prime }}\left(A_{i}\right)\;\;\mathrm{if}\;an_{G^{\prime }}\left(A_{i}\right)⊥an_{G^{\prime }}\left(Z_{j}\right)\cap an_{G^{\prime }}\left(A_{\left[n\right]}\right)|Z\\ an_{G^{\prime }}\left(A_{i}\right)\cup \left(an_{G^{\prime }}\left(Z_{j}\right)\cap an_{G^{\prime }}\left(A_{\left[n\right]}\right)\right)\;\;\mathrm{otherwise}. \end{array}\right.$$

We then define the set $S\left(G^{\prime };Z_{j}\right)\equiv \left\{A_{i}\in A_{\left[n\right]}:an_{G^{\prime }}\left(A_{i}\right)an_{G^{\prime }}\left(Z_{j}\right)\cap an_{G^{\prime }}\left(A_{\left[n\right]}\right)|Z\right\}$, that is, the set of groups that have some ancestor not independent from some ancestor of $Z_{j}$ that is also ancestor of $A_{\left[n\right]}$, given Z.

For each $A_{i}$, let $d_{i}\left(G^{\prime };Z_{j}\right)$ be the maximal number such that a non-empty intersection exists between $an_{G^{\prime }}\left(A_{i};Z_{j}\right)$ and $d_{i}\left(G^{\prime };Z_{j}\right)-1$ other distinct augmented ancestral sets of $A_{i_{1}},\cdots ,A_{i_{d_{i}\left(G^{\prime };Z_{j}\right)-1}}$. Furthermore, we define $d_{i}\left(G^{\prime };Z\right)$ as the maximum for all $Z_{j}\in Z$:

(8) $$d_{i}\left(G^{\prime };Z\right)\equiv \max_{Z_{j}\in Z}d_{i}\left(G^{\prime };Z_{j}\right).$$

We will use $d(G^{\prime };Z)$ to refer to the whole set of maximal values for all groups. If required, we will use $d_{A_{\left[n\right]}}\left(G^{\prime };Z\right)$ to specify that the collection is $A_{\left[n\right]}$. 

**Figure 4:**
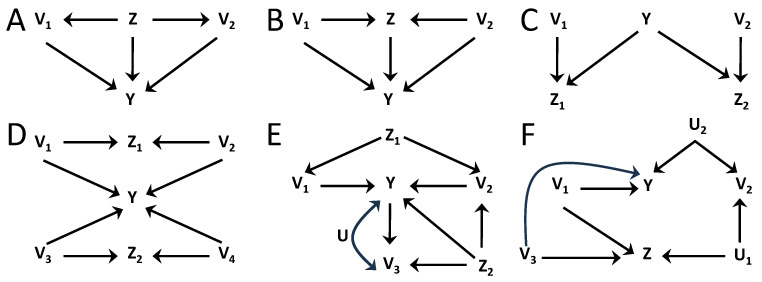
Inequalities involving sums of information terms from ancestral sets. (**A**–**D**) Examples to illustrate the definition of augmented ancestral sets (Equations (7) and (8)). (**E**,**F**) Examples of the application of Theorem 2 to obtain testable inequalities.

In Figure 4A–D, we consider examples to understand the rationale of how $d_{A_{\left[n\right]}}\left(G^{\prime };Z\right)$ is determined in inequalities with a conditioning Z. In Figure 4A, for groups $A_{1}=\left\{V_{1}\right\}$ and $A_{2}=\left\{V_{2}\right\}$, the augmented ancestral sets on graph *G* are $an_{G}\left(A_{1};Z\right)=\left\{V_{1},Z\right\}$ and $an_{G}\left(A_{2};Z\right)=\left\{V_{2},Z\right\}$, which intersect on *Z* and $d_{i}\left(G;Z\right)=2$ for i=1,2. However, *Z* is a noncollider in the path creating a dependence between $V_{1}$ and $V_{2}$, and conditioning on *Z* renders them independent, so that $d_{i}\left(G;Z\right)=2$ overestimates the amount of information the groups may share after conditioning. Alternatively, selecting $G_{\underline{Z}}$ the ancestral sets are $an_{G_{\underline{Z}}}\left(A_{1};Z\right)=\left\{V_{1}\right\}$ and $an_{G_{\underline{Z}}}\left(A_{2};Z\right)=\left\{V_{2}\right\}$, which do not intersect and $d_{i}\left(G_{\underline{Z}};Z\right)=1$ for i=1,2 when calculated following Equation (7). A priori, we do not know which graph $G^{\prime }=G_{\underline{Z^{\prime }}}$, $Z^{\prime }\subseteq Z$, results in a tighter inequality. Here we see that $G_{\underline{Z}}$ leads to an inequality with more causal inference power than *G* for Figure 4A. In Figure 4B, *Z* is a collider between $V_{1}$ and $V_{2}$, so that conditioning on *Z* creates a dependence between the groups. If the values $d_{i}$ were determined from the standard ancestral sets, in this case $an_{G}\left(A_{i}\right)=an_{G_{\underline{Z}}}\left(A_{i}\right)=\left\{V_{i}\right\}$, for i=1,2, which do not intersect, leading to unit coefficients. However, the augmented ancestral sets following Equation (7) are $an_{G}\left(A_{i};Z\right)=an_{G_{\underline{Z}}}\left(A_{i};Z\right)=\left\{V_{1},V_{2}\right\}$ for i=1,2, so that $d_{i}\left(G;Z\right)=d_{i}\left(G_{\underline{Z}};Z\right)=2$. This illustrates that the augmented ancestral sets are necessary to properly determine the coefficients in inequalities with conditioning sets Z, in this case reflecting that $I(Y;V_{1}|Z)$ and $I(Y;V_{2}|Z)$ can have redundant information.

Figure 4C shows a scenario in which conditioning creates dependencies of *Y* with $V_{1}$ and $V_{2}$, which were previously independent. The standard ancestral sets $an_{G^{\prime }}\left(A_{1}\right)=\left\{V_{1}\right\}$ and $an_{G^{\prime }}\left(A_{2}\right)=\left\{V_{2}\right\}$ would not intersect in any $G^{\prime }=G_{\underline{Z^{\prime }}}$, with $Z^{\prime }\subseteq \left\{Z_{1},Z_{2}\right\}$ and would lead to unit values for $d_{i}$. On the other hand, the augmented ancestral sets are $an_{G^{\prime }}\left(A_{i};Z_{j}\right)=\left\{V_{i}\right\}$ for i=j and $an_{G^{\prime }}\left(A_{i};Z_{j}\right)=\left\{V_{1},V_{2}\right\}$ for i≠j, for all $G^{\prime }=G_{\underline{Z^{\prime }}}$, with $Z^{\prime }\subseteq \left\{Z_{1},Z_{2}\right\}$. This results in $d_{i}\left(G^{\prime };Z\right)=2$ in all cases, which appropriately captures that the two groups can have common information about *Y* when conditioning on $\left\{Z_{1},Z_{2}\right\}$. The example of Figure 4D illustrates why each value $d_{i}\left(G^{\prime };Z_{j}\right)$ is determined separately (Equation (7)) first, and only after is the maximum calculated (Equation (8)). Four groups are defined as $A_{i}=V_{i}$ for i=1,⋯,4. If $d_{i}\left(G^{\prime };Z\right)$ were to be determined directly from Equation (7) but using $Z=\{Z_{1},Z_{2}\}$, instead of using separately $Z_{1}$ and $Z_{2}$, then for all the ancestral sets the augmented ancestral set would include all variables, since $an_{G^{\prime }}\left(Z\right)\cap an_{G^{\prime }}\left(A_{\left[n\right]}\right)$ is equal to $an_{G^{\prime }}\left(A_{\left[n\right]}\right)$. This would lead to $d_{i}=4,\forall i$. However, that determination would overestimate how many groups become dependent when conditioning on Z, since $Z_{1}$ creates a dependence between $V_{1}$ and $V_{2}$ and $Z_{2}$ between $V_{3}$ and $V_{4}$, but no dependencies across these pairs are created. The determination of $d(G^{\prime };Z)=2$ from Equations (7) and (8) properly leads to a tighter inequality than the one obtained if considering jointly both conditioning variables.

Equipped with this extended definition of $d_{A_{\left[n\right]}}\left(G^{\prime };Z\right)$, we now present our generalization of Theorem 1:

**Theorem 2.**  *Let G be a DAG model containing nodes corresponding to a set of (possibly hidden) variables X. Let Y∈X be a set of observable target variables, and $Z=\{Z_{1},\cdots ,Z_{m}\}$ a conditioning set of observable variables, with Z⊂X. Let $A_{\left[n\right]}=\left\{A_{1},\cdots ,A_{n}\right\}$ be a collection of (possibly overlapping) groups of (possibly hidden) variables $A_{i}\subset X$. Consider a DAG $G^{\prime }$ selected as $G^{\prime }=G_{\underline{Z^{\prime }}}$ with $Z^{\prime }\subseteq Z$, constructed by removing from graph G all the outgoing arrows from nodes in $Z^{\prime }$. Following Equation (7), define an augmented ancestral set in $G^{\prime }$ for each group $A_{i}\in A_{\left[n\right]}$ for each variable in the conditioning set, $Z_{j}\in Z$. Following Equation (8), determine $d_{i}\left(G^{\prime };Z\right)$ for each group, given the intersections of the augmented ancestral sets $an_{G^{\prime }}\left(A_{i};Z_{j}\right)$. Select a variable $W_{0}\in an_{G^{\prime }}\left(A_{\left[n\right]}\right)$ and a group of variables $W\subseteq D_{G^{\prime }}\left(W_{0}\right)\cap an_{G^{\prime }}\left(A_{\left[n\right]}\right)$, possibly W=∅. Define the reduced ancestral sets ${\tilde{an}}_{G^{\prime }}\left(A_{i}\right)\equiv an_{G^{\prime }}\left(A_{i}\right)\setminus W$ for each $A_{i}\in A_{\left[n\right]}$, and the reduced collection ${\tilde{an}}_{G^{\prime }}\left(A_{\left[n\right]}\right)\equiv an_{G^{\prime }}\left(A_{\left[n\right]}\right)\setminus W$. The information about Y in this reduced collection when conditioning on Z is bounded from below by*

(9) $$I\left(Y;{\tilde{an}}_{G^{\prime }}\left(A_{\left[n\right]}\right)|Z\right)\geq \sum_{i=1}^{n}\frac{1}{d_{i}\left(G^{\prime };Z\right)}I\left(Y;{\tilde{an}}_{G^{\prime }}\left(A_{i}\right)|Z\right).$$

**Proof.**  The proof is provided in Appendix B. □

Theorem 2 provides several extensions of Theorem 1. First, it allows for a conditioning set Z. Second, given a hypothesis of the generative causal graph *G* underlying the data, Theorem 2 can be applied to any $G^{\prime }=G_{\underline{Z^{\prime }}}$ with $Z^{\prime }\subseteq Z$, and hence offers a set of inequalities potentially adding causal inference power. As we have discussed in relation to Figure 4A–D, the selection of $G^{\prime }$ that leads to the tightest inequality in some cases will be determined by the causal structure, but in general it also depends on the exact probability distribution of the variables. Third, Theorem 2 allows excluding some variables W from the ancestral sets, although imposing constraints in the causal structure of W. The role of these constraints is clear in the proof at Appendix B. The case of Theorem 1 corresponds to Z=∅, W=∅, and $G^{\prime }=G$.

Excluding some variables W can be advantageous. For example, if Y is univariate and it overlaps with some ancestral sets, as it is the case when some groups include descendants of *Y*, then the upper bound $I(Y;an_{G^{\prime }}\left(A_{\left[n\right]}\right)|Z)$ is equal to H(Y|Z) and also $I(Y;an_{G^{\prime }}\left(A_{i}\right)|Z)$ is equal to H(Y|Z) for all ancestral sets that include *Y*. Excluding W=Y provides a tighter upper bound $I(Y;an_{G^{\prime }}\left(A_{\left[n\right]}\right)\setminus Y|Z)$ and may provide more causal inferential power. Another scenario in which a reduced collection can be useful is when excluding W removes all hidden variables from $an_{G^{\prime }}\left(A_{\left[n\right]}\right)$, such that ${\tilde{an}}_{G^{\prime }}\left(A_{\left[n\right]}\right)$ is observable, giving $I(Y;{\tilde{an}}_{G^{\prime }}\left(A_{\left[n\right]}\right)|Z)$ as a testable upper bound instead of H(Y|Z). When comparing inequalities with different sets W, in some cases the form of the causal structure and the specification of which variables are hidden or observable will a priori determine an order of causal inference power among the inequalities. However, like for the comparison across $G^{\prime }=G_{\underline{Z^{\prime }}}$ with $Z^{\prime }\subseteq Z$, in general the power of the different inequalities depends on the details of the generated probability distributions. Formulating general criteria to rank inequalities with different Z, $G^{\prime }$, and W in terms of their inferential power is beyond the scope of this work.

Note that we have formulated Theorem 2 explicitly allowing for hidden variables. Also, in Theorem 1 (as a subcase of Theorem 2) the restriction of $A_{\left[n\right]}$ being observable variables can be removed. In any case, the inclusion of hidden variables can only increase the causal inference power if combined with data processing inequalities to obtain a testable inequality. Propositions 4–6 indicate how to possibly tighten an inequality derived from Proposition 1 by substituting $A_{\left[n\right]}$ by a new collection $B_{\left[n\right]}$ that, including hidden variables, leads to $d_{B_{\left[n\right]}}$ smaller than $d_{A_{\left[n\right]}}$. The same application of data processing inequalities of the unique and conditional mutual information can be used for Theorem 2 to determine a $B_{\left[n\right]}$ with $d_{B_{\left[n\right]}}\left(G^{\prime };Z\right)$ smaller than $d_{A_{\left[n\right]}}\left(G^{\prime };Z\right)$. The use of data processing inequalities is necessary because they allow substituting some of the observable variables by hidden variables, instead of only adding hidden variables. When only adding variables, the number of intersections between ancestral groups can only increase, hence not decreasing $d(G^{\prime };Z)$. On top of this, a testable inequality replaces information terms of ancestral groups by their lower bounds given by observable subsets of variables. This means that, adding hidden variables, the testable inequality will contain the same information terms of the observable variables, but possibly smaller coefficients, hence resulting in a looser inequality. This is not the case any more when hidden variables are not added but instead substitute some of observable variables, thanks to data processing inequalities. This substitution may decrease the number of intersections between ancestral groups, and the coefficients in the sum may be higher. We will not describe this procedure in detail, since the use of data processing inequalities is analogous to their use in Propositions 4–6.

We now illustrate the application of Theorem 2. In Figure 4E, with $Z=\{Z_{1},Z_{2}\}$, the conditions of independence required by Proposition 6 do not hold for any set of groups, either $A_{i}=\left\{V_{i}\right\}$, i=1,2,3, or, with i≠j≠k, $A_{1}=\left\{V_{i},V_{j}\right\}$, $A_{2}=\left\{V_{i},V_{k}\right\}$ or $A_{1}=\left\{V_{i},V_{j}\right\}$, $A_{2}=\left\{V_{k}\right\}$. No data processing inequalities can be applied to replace some variables to fulfill the conditions. On the other hand, Theorem 2 can always be applied, since it does not require the fulfillment of some conditions of independence. For example, for $A_{i}=\left\{V_{i}\right\}$, i=1,2,3 and for $G^{\prime }=G_{\underline{Z_{1}Z_{2}}}$, we have $an_{G^{\prime }}\left(V_{1}\right)=\left\{V_{1}\right\}$, $an_{G^{\prime }}\left(V_{2}\right)=\left\{V_{2}\right\}$, $an_{G^{\prime }}\left(V_{3}\right)=\left\{V_{1},V_{2},V_{3},U,Y\right\}$, and following Equation (7) $an_{G^{\prime }}\left(V_{1};Z_{j}\right)=\left\{V_{1}\right\}$, $an_{G^{\prime }}\left(V_{2};Z_{j}\right)=\left\{V_{2}\right\}$, and $an_{G^{\prime }}\left(V_{3};Z_{j}\right)=\left\{V_{1},V_{2},V_{3},U,Y\right\}$, for j=1,2. This leads to $d\left(G^{\prime };Z\right)=\left\{2,2,3\right\}$. For illustration purpose, we focus on W equal to {Y,U} or any of its subsets. In all cases ${\tilde{an}}_{G^{\prime }}\left(V_{i}\right)=an_{G^{\prime }}\left(V_{i}\right)$, for i=1,2, contributing terms $1/2I(Y;V_{1}|Z_{1},Z_{2})$ and $1/2I(Y;V_{2}|Z_{1},Z_{2})$. For W={Y,U} or W={Y}, the contribution of the observable lower bound of the third group is $1/3I(Y;V_{1},V_{2},V_{3}|Z_{1},Z_{2})$. For W={U} or W=∅, the third group contributes $1/3H(Y|Z_{1},Z_{2})$. For W={Y,U}, ${\tilde{an}}_{G^{\prime }}\left(A_{\left[n\right]}\right)=\left\{V_{1},V_{2},V_{3}\right\}$, which is observable and the upper bound is $I(Y;V_{1},V_{2},V_{3}|Z_{1},Z_{2})$. For any other subset of {Y,U} the upper bound in the testable inequality is $H(Y|Z_{1},Z_{2})$. Because the terms in the sum for groups 1 and 2 are equal for all the W compared, in this case it can be checked that selecting W={Y,U} leads to the tightest inequality. This example illustrates the utility of being able to construct inequalities for reduced ancestral sets.

While in the previous example only Theorem 2 and not Proposition 6 was applicable, more generally, a causal structure will involve the fulfillment of a set of inequalities, some obtained using Proposition 6 and some using Theorem 2. Which inequalities have higher inferential power will depend on the causal structure and the exact probability distribution of the data, and neither Theorem 2 nor Proposition 6 are more powerful a priori. In Figure 4F, Proposition 6 cannot be applied using $A_{i}=\left\{V_{i}\right\}$, i=1,2,3 and conditioning on *Z*, because $V_{i}$
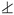
$V_{j}|Z$, ∀i,j and no data processing inequalities help to substitute these variables. On the other hand, Theorem 2 can be applied with $A_{i}=\left\{V_{i}\right\}$, leading to $an_{G^{\prime }}\left(V_{1}\right)=\left\{V_{1}\right\}$, $an_{G^{\prime }}\left(V_{3}\right)=\left\{V_{3}\right\}$, and $an_{G^{\prime }}\left(V_{2}\right)=\left\{V_{2},U_{1},U_{2}\right\}$, for all $G^{\prime }=G_{\underline{Z^{\prime }}}$. The augmented ancestral sets are $an_{G^{\prime }}\left(V_{1};Z\right)=\left\{V_{1},V_{3},U_{1}\right\}$$=an_{G^{\prime }}\left(V_{3};Z\right)$, and $an_{G^{\prime }}\left(V_{2};Z\right)=\left\{V_{1},V_{2},V_{3},U_{1},U_{2}\right\}$, also for all $G^{\prime }$, resulting in $d(G^{\prime };Z)=3$. Focusing on the case of $W=\{Y,U_{2}\}$, or any subset of it, in all cases the associated testable inequality has H(Y|Z) as upper bound and in the r.h.s. the sum of terms $1/3I(Y;V_{i}|Z)$, i=1,2,3. Alternatively, defining $A_{1}=\left\{V_{1},V_{3},U_{1}\right\}$ and $A_{2}=\left\{V_{2},U_{1}\right\}$, Proposition 3 is applicable with the two groups intersecting in $U_{1}$ and $V_{1},V_{3}⊥V_{2}|Z,U_{1}$. The associated testable inequality has the same upper bound H(Y|Z) and in the r.h.s. the sum of terms $1/2I(Y;V_{1},V_{3}|Z)$ and $1/2I(Y;V_{2}|Z)$. In this case, which inequality has more causal inferential power will depend on the exact distribution of the data.

Overall, Theorem 2 extends Theorem 1 of [27], allowing conditioning sets and providing more flexible conditions to form the groups. In the examples of Figure 4, we have illustrated how Theorem 2 substantially increases the number of groups-decomposition inequalities that can be tested to reject hypothesized causal structures to be compatible with a certain data set.

## 4. Discussion

We have presented several generalizations of the type of groups-decomposition inequalities introduced by [27], which compare the information about target variables contained in a collection of variables with a weighted sum of the information contained in subsets of the collection. These generalizations include an extension to allow for conditioning sets and methods to identify existing inequalities that involve collections and subsets selected with less restrictive criteria. This comprises less restrictive conditions of independence, the use of ancestral sets from subgraphs of the causal structure of interest, and the removal of some variables from the ancestral sets. We have also shown how to exploit inequalities identified for collections containing hidden variables—which are not directly testable—by converting them into testable inequalities using data processing inequalities.

Our use of data processing inequalities to derive testable groups-decomposition inequalities when collections contain hidden variables is not entirely new. We found inspiration for this approach in the proof of Theorem 1 in [24]. This theorem derives a causally informative inequality from a particular type of causal structure, namely common ancestor graphs in which all dependencies between observable variables are caused by hidden common ancestors. The inequality presented in the theorem corresponds to the setting of a univariate target variable and groups composed by different single observable variables. In their simplest case, each hidden ancestor only has two children, which are observable variables. Their proof uses the mutual information data processing inequality to convert a sum of information terms involving the observable variables into a sum of terms involving the hidden ancestors. The final inequality can equally be proven applying our Proposition 4 by deriving an inequality for the collection of hidden variables and then converting it into a testable inequality using data processing inequalities. The same final inequality can also be derived as an application of our Theorem 2 followed by the use of the data processing inequality.

We have expanded the applicability of data processing inequalities by showing that this type of inequality also holds for conditional unique information measures [29]. For a given causal structure, a testable causally informative inequality may be obtained substituting hidden variables by observable variables thanks to the data processing inequality of the unique information, in cases in which the data processing inequality of mutual information is not applicable. As shown in Proposition 6, the unique information data processing inequalities are particularly powerful for deriving groups-decomposition inequalities with a conditioning set, since they can iteratively be applied to replace different subsets of hidden variables by observable variables choosing which variables are kept as conditioning variables and which ones are taken as reference variables for different unique information measures. This use of unique information indicates how other types of information-theoretic measures could be similarly incorporated to derive causally informative inequalities. Recent developments in the decomposition of mutual information into redundant, synergistic, and unique contributions [30] provide candidate measures whose utility for this purpose needs to be further explored [31,32,33,34,35,40,41] (among others). Furthermore, while this type of decomposition has been extensively debated recently [35,42,43], aspects of its characterization are still unsolved and an understanding of how the terms are related to the causal structure can provide new insights.

One particular domain in which our generalizations can be useful is to study causal interactions among dynamical processes [23,44], for which causal interactions are characterized from time series both in the temporal [45] and spectral domain [46,47,48]. When studying high-dimensional multivariate dynamical processes, such as brain dynamics (e.g., [49,50,51]) or econometric data [52,53], an important question is to determine whether correlations between time series are related to causal influences or to hidden common influences. For highly interconnected systems with many hidden variables, the number of independencies may be small, hence providing limited information about the causal structure. In this case, inequality constraints can help to substantially narrow down the set of causal structures compatible with the data. Accordingly, our generalization to formulate conditional inequalities may play an important role in combination with measures to quantify partial dependencies between time series [54,55]. We expect this approach to be easily adaptable to non-stationary time-series, as it is often the case in the presence of unit roots and co-integrated time series [56,57,58]. This can be carried out by selecting collections and groups consistent with the temporal partitioning in non-stationary information-theoretic measures of causality in time-series [59,60]. Another area to extend the applicability of our proposal is to study non-classical quantum systems [16,61,62,63]. In this case, an extended d-separation criterion [64] and adapted faithfulness considerations [65] have been proposed to take into account the particularities of quantum systems. Further exploration will be required to determine if and how our derivations that rely on d-separation leading to statistical independence (Appendix C) are also applicable when considering generalized causal structures for quantum systems.

Besides the extension to particular domains, an important question yet to be addressed regards the relation between the causal inferential power of different inequalities. Our proposal considerably enlarges the number of groups-decomposition inequalities of the type of [27] available to test the compatibility of a causal structure with a given data set. We have seen in our analysis some examples of how, under certain conditions, the causal structure imposes an ordering to the power of alternative inequalities. Future work should aim to derive broader criteria to rank the inferential power of inequalities, for example in terms of the relation between the conditioning sets or the constituency of the groups that appear in each inequality. Formulating criteria to rank the inferential power of different inequalities would help to simplify the set of inequalities that needs to be tested when the compatibility of a certain causal structure with the data is to be examined.

Apart from a characterization of how groups-decomposition inequalities are related among themselves, future work should also examine the relation and embedding of this type of inequalities with those derived with other approaches. In our understanding, the algorithmic projection procedure of [23,24] could equally retrieve some of the inequalities here described, but without the advantage of having a constructive procedure to derive the form of an inequality directly reading a causal graph, and instead requiring costly computations that may limit the derivation of inequalities for large systems. The incorporation of constraints for other types of information-theoretic measures, such as constraints involving unique information measures, would require an extension of the algorithmic approach. Among other approaches, the so-called *Inflation technique* [66] stands out as capable of providing asymptotically sufficient tests of causal compatibility [67]. The inflation method creates a new causal structure with multiple copies of the original structure and symmetry constraints on the ancestral properties of the different copies, in such a way that testable constraints on the inflated graph can be mapped back to the compatibility of the original causal structure. However, despite the ongoing advances in its theoretical developments and implementation [68], to our knowledge it is not straightforward to identify the order of inflation and the specific inflation structure adequate to discriminate between certain causal structures. The availability of inequalities easily derived by reading the original causal structure can also be helpful in combination with the inflation method, in order to discard as many candidate causal structures as possible before the design of additional inflated graphs. The connection with other approaches [69,70,71,72,73,74] also deserves further investigation, ultimately to determine minimal sets of inequality constraints with equivalent inferential power.

Beyond the derivation of existing testable causally informative inequalities, a crucial issue for their application is the implementation of the corresponding tests. This implementation depends on the estimation of information-theoretic measures from data. A ubiquitous challenge for the application of mutual information measures is that they are positively biased and their estimation is data-demanding [75,76]. These biases scale with the dimensionality of the variables, and hence can hinder the applicability of information-theoretic inequalities for large collections of variables, or for variables with high cardinality. However, recent advances in the estimation of mutual information for high-dimensional data can help to attenuate these biases [77]. Furthermore, the implementation of the tests can take advantage of the existence of both upper-bound and lower-bound estimators of mutual information [78], using opposite bounds at the two sides of the inequalities. These technical aspects of the implementation of the tests are important to apply all types of information-theoretic inequalities [23,24,25,26,27,71]. Despite these common challenges, our extension of groups-decomposition inequalities does not come at the price of having to test inequalities that intrinsically are more difficult to estimate. Our contribution can substantially increase the number of inequalities available to be tested, and we have provided examples in Figure 2, Figure 3 and Figure 4 of new inequalities in which—in particular thanks to the use of data processing inequalities—the dimensionality of the collections is not increased. Future work is required to determine how to efficiently combine all available tests. In the goal to determine minimal sets of inequality tests that are maximally informative, the statistical power of the tests will need to be considered together with their discrimination power among causal structures.

## Appendix A. Proofs of Propositions 1, 3, and 6

**Proof** **of Proposition 1** Given a collection $A_{\left[n\right]}=\left\{A_{1},\cdots ,A_{n}\right\}$, define $X_{\left[r\right]}$ as the set of *r* variables that are part of at least a group in $A_{\left[n\right]}$. We have that

(A1) $$\begin{array}{cc} \; & I\left(Y;A_{\left[n\right]}|Z\right)\overset{\left(a\right)}{=}I\left(Y;X_{\left[r\right]}|Z\right)\overset{\left(b\right)}{=}\sum_{k=1}^{r}I\left(Y;X_{k}|X_{\left[k-1\right]},Z\right)\overset{\left(c\right)}{\geq }\\ \; & \sum_{k=1}^{r}I\left(Y;X_{k}|X_{\left[k-1\right]},Z\right)\left(\sum_{A_{i}:X_{k}\in A_{i}}\frac{1}{d_{i}}\right)\overset{\left(d\right)}{=}\sum_{i=1}^{n}\frac{1}{d_{i}}\sum_{X_{k}\in A_{i}}I\left(Y;X_{k}|X_{\left[k-1\right]},Z\right)\overset{\left(e\right)}{\geq }\\ \; & \sum_{i=1}^{n}\frac{1}{d_{i}}\sum_{X_{k}\in A_{i}}I\left(Y;X_{k}|\left(X_{\left[k-1\right]}\cap A_{i}\right),Z\right)\overset{\left(f\right)}{=}\sum_{i=1}^{n}\frac{1}{d_{i}}I\left(Y;A_{i}|Z\right) \end{array}$$

Equality (a) follows from $X_{\left[r\right]}$ containing the same variables as $A_{\left[n\right]}$. Equality (b) follows from the iterative application of the chain rule for mutual information, where $X_{\left[0\right]}\equiv \emptyset$ and $X_{\left[k-1\right]}=\left\{X_{0},\cdots ,X_{k-1}\right\}$. Inequality (c) follows from the definition of $d_{i}$ as maximal, such that the number of groups that contain $X_{k}$ is equal or smaller than $d_{i}$ for all $A_{i}$ containing $X_{k}$, and hence $\sum_{A_{i}:X_{k}\in A_{i}}1/d_{i}\leq 1$. Equality (d) groups together into the inner sum variables within the same group. Inequality (e) follows from Lemma 1(ii). In more detail, $A_{i}⊥A_{j}\setminus A_{i}|Z\;\forall i,j$, combined with the weak union property of independencies [27], ensures that for each $X_{k}\in A_{i}$, $X_{k}⊥\left(X_{\left[k-1\right]}\cap A_{j}\right)\setminus A_{i}|\left(X_{\left[k-1\right]}\cap A_{i}\right),Z$, ∀j≠i. Assuming faithfulness, this implies $X_{k}⊥X_{\left[k-1\right]}\setminus A_{i}|\left(X_{\left[k-1\right]}\cap A_{i}\right),Z$. Lemma 1(ii) applies with $A=X_{k}$, $B=\{\left(X_{\left[k-1\right]}\cap A_{i}\right),Z\}$, and $C=X_{\left[k-1\right]}\setminus A_{i}$. Equality (f) follows applying the chain rule within each group $A_{i}$. □

Proposition 1 of [27] is included in the case Z=∅. The faithfulness assumption allows relaxing their assumption $X_{k}⊥X_{\left[r\right]}\setminus X_{k}\forall k$ to $A_{i}⊥A_{j}\setminus A_{i}|Z$∀i,j. A tighter bound can be obtained in some cases if some variables are trimmed. In particular, for a variable $X^{\prime }$, $A_{j}$ can be trimmed to $A_{j}\setminus X^{\prime }$ for all groups such that $I\left(Y;A_{j}|Z\right)=I(Y;A_{j}\setminus X^{\prime }\left|Z\right)$ and possibly lower $d_{j}$ values can be obtained after trimming. We do not explicitly include this trimming process in the definition of $d_{j}$ to simplify the formulation.

**Proof** **of Proposition 3** Consider a collection of groups $B_{\left[n\right]}=\left\{B_{1},\cdots ,B_{n}\right\}$, each with a partition in disjoint subsets $B_{i}=\left\{B_{i}^{\left(1\right)},B_{i}^{\left(2\right)}\right\}$ that fulfill the conditions $B_{i}^{\left(1\right)}⊥B_{j}^{\left(1\right)}\setminus B_{i}^{\left(1\right)}|Z$ and $B_{i}^{\left(2\right)}⊥B_{j}\setminus B_{i}^{\left(2\right)}|B_{i}^{\left(1\right)}Z$∀i,j, and such that $B_{\left[n\right]}^{\left(1\right)}=\left\{B_{1}^{\left(1\right)},\cdots ,B_{n}^{\left(1\right)}\right\}$ and $B_{\left[n\right]}^{\left(2\right)}=\left\{B_{1}^{\left(2\right)},\cdots ,B_{n}^{\left(2\right)}\right\}$ are disjoint. Define $X_{\left[r_{k}\right]}^{\left(k\right)}$ as the set of $r_{k}$ variables part of at least a group in $B_{\left[n\right]}^{\left(k\right)}$, for k=1,2. We have that

(A2) $$\begin{array}{cc} \; & I\left(Y;B_{\left[n\right]}|Z\right)\overset{\left(a\right)}{=}I\left(Y;X_{\left[r_{1}\right]}^{\left(1\right)},X_{\left[r_{2}\right]}^{\left(2\right)}|Z\right)\overset{\left(b\right)}{=}\\ \; & \sum_{X_{k}\in X_{\left[r_{1}\right]}^{\left(1\right)}}I\left(Y;X_{k}|X_{\left[k-1\right]}^{\left(1\right)},Z\right)+\sum_{X_{k}\in X_{\left[r_{2}\right]}^{\left(2\right)}}I\left(Y;X_{k}|X_{\left[k-1\right]}^{\left(2\right)},X_{\left[r_{1}\right]}^{\left(1\right)},Z\right)\overset{\left(c\right)}{\geq }\\ \; & \sum_{i=1}^{n}\frac{1}{d_{i}}I\left(Y;B_{i}^{\left(1\right)}|Z\right)+\sum_{i=1}^{n}\frac{1}{d_{i}}\sum_{X_{k}\in B_{i}^{\left(2\right)}}I\left(Y;X_{k}|X_{\left[k-1\right]}^{\left(2\right)},X_{\left[r_{1}\right]}^{\left(1\right)},Z\right)\overset{\left(d\right)}{\geq }\\ \; & \sum_{i=1}^{n}\frac{1}{d_{i}}I\left(Y;B_{i}^{\left(1\right)}|Z\right)+\sum_{i=1}^{n}\frac{1}{d_{i}}\sum_{X_{k}\in B_{i}^{\left(2\right)}}I\left(Y;X_{k}|\left(X_{\left[k-1\right]}^{\left(2\right)}\cap B_{i}^{\left(2\right)}\right),B_{i}^{\left(1\right)},Z\right)\overset{\left(e\right)}{=}\\ \; & \sum_{i=1}^{n}\frac{1}{d_{i}}I\left(Y;B_{i}^{\left(1\right)}|Z\right)+\sum_{i=1}^{n}\frac{1}{d_{i}}I\left(Y;B_{i}^{\left(2\right)}|B_{i}^{\left(1\right)}Z\right)\overset{\left(f\right)}{=}\sum_{i=1}^{n}\frac{1}{d_{i}}I\left(Y;B_{i}|Z\right). \end{array}$$

Equality (a) holds because $\left\{X_{\left[r_{1}\right]}^{\left(1\right)},X_{\left[r_{2}\right]}^{\left(2\right)}\right\}$ contains the same variables as $B_{\left[n\right]}$. Equality (b) is an iterative application of the chain rule. Inequality (c) is as follows: For the sum in $X_{\left[r_{1}\right]}^{\left(1\right)}$, steps (c) to (f) of Equation (A1) are all combined, substituting sets $A_{i}$ by $B_{i}^{\left(1\right)}$ and given that these variables fulfill conditions of independence equivalent to Proposition 1. For the sum in $X_{\left[r_{2}\right]}^{\left(2\right)}$, only steps (c) and (d) of Equation (A1) are applied, substituting sets $A_{i}$ by $B_{i}^{\left(2\right)}$. Inequality (d) holds applying Lemma 1(ii). In more detail, $B_{i}^{\left(2\right)}⊥B_{j}\setminus B_{i}^{\left(2\right)}|B_{i}^{\left(1\right)}Z$∀i,j combined with the weak union property of independencies [27] mean that for each $X_{k}\in B_{i}^{\left(2\right)}$, $X_{k}⊥\left\{B_{j}^{\left(1\right)},\left(X_{\left[k-1\right]}^{\left(2\right)}\cap B_{j}^{\left(2\right)}\right)\setminus B_{i}^{\left(2\right)}\right\}|\left(X_{\left[k-1\right]}^{\left(2\right)}\cap B_{i}^{\left(2\right)}\right),B_{i}^{\left(1\right)},Z$∀j≠i. Assuming faithfulness, this implies $X_{k}⊥\left\{\left(X_{\left[r_{1}\right]}^{\left(1\right)}\setminus B_{i}^{\left(1\right)}\right),\left(X_{\left[k-1\right]}^{\left(2\right)}\setminus B_{i}^{\left(2\right)}\right)\right\}|\left(X_{\left[k-1\right]}^{\left(2\right)}\cap B_{i}^{\left(2\right)}\right),B_{i}^{\left(1\right)},Z$. Accordingly, Lemma 1 (ii) applies with $A=X_{k}$, $B=\{\left(X_{\left[k-1\right]}^{\left(2\right)}\cap B_{i}^{\left(2\right)}\right),B_{i}^{\left(1\right)},Z\}$, and $C=\{\left(X_{\left[r_{1}\right]}^{\left(1\right)}\setminus B_{i}^{\left(1\right)}\right),\left(X_{\left[k-1\right]}^{\left(2\right)}\setminus B_{i}^{\left(2\right)}\right)\}$. Equalities (e) and (f) follow from the chain rule of mutual information. □

Before continuing with the proof of Proposition 6, we formulate in Lemma A1 a property of the unique information that will be used in the proof.

**Theorem A1.** 
*(Conditioning on reference variables increases conditional unique information): The conditional unique information $I(Y;X\setminus \setminus Z_{1}Z_{2}|Z_{3})$ is smaller than or equal to $I(Y;X\setminus \setminus Z_{1}|Z_{2}Z_{3})$, where $Z_{2}$ moves from the set of reference predictors of the unique information to the conditioning set.*

**Proof of Lemma A1.** The unique information $I(Y;X\setminus \setminus Z_{1}Z_{2}|Z_{3})$ is by definition (Equation (2)) the minimum information $I(Y;X|Z_{1}Z_{2}Z_{3})$ among the distributions that preserve $P(Y,X,Z_{3})$ and $P(Y,Z_{1},Z_{2},Z_{3})$, and $I(Y;X\setminus \setminus Z_{1}|Z_{2}Z_{3})$ is the minimum information $I(Y;X|Z_{1}Z_{2}Z_{3})$ among the distributions that preserve $P(Y,X,Z_{2},Z_{3})$ and $P(Y,Z_{1},Z_{2},Z_{3})$. Since the latter constraints subsume the former ones, the minimum can only be equal or higher. □

**Proof of Proposition 6.** For iterations $k=1,...,m_{i}-1$, consider the following:

(A3) $$\begin{array}{cc} \; & I(Y;C_{i}^{\left[k-1\right]}A_{i}\setminus A_{i}^{\left[k-1\right]}\setminus \setminus Z\setminus Z_{i}^{\left[k-1\right]}|Z_{i}^{\left[k-1\right]})\overset{\left(a\right)}{\leq }I(Y;C_{i}^{\left[k\right]}A_{i}\setminus A_{i}^{\left[k-1\right]}\setminus \setminus Z\setminus Z_{i}^{\left[k-1\right]}\left|Z_{i}^{\left[k-1\right]}\right)\overset{\left(b\right)}{\leq }\\ \; & I(Y;C_{i}^{\left[k\right]}A_{i}\setminus A_{i}^{\left[k-1\right]}\setminus \setminus Z\setminus Z_{i}^{\left[k\right]}|Z_{i}^{\left[k\right]})\overset{\left(c\right)}{=}I(Y;C_{i}^{\left[k\right]}A_{i}\setminus A_{i}^{\left[k\right]}\setminus \setminus Z\setminus Z_{i}^{\left[k\right]}\left|Z_{i}^{\left[k\right]}\right). \end{array}$$

Inequality (a) holds from monotonicity, information cannot decrease if adding $C_{i}^{\left(k\right)}$ to $C_{i}^{\left[k-1\right]}$. Inequality (b) holds from Lemma A1, moving $Z_{i}^{\left(k\right)}$ from the set of reference predictors of the unique information to the conditioning set. Equality (c) follows from $A_{i}\setminus A_{i}^{\left[k-1\right]}=$$\left\{A_{i}^{\left(k\right)},A_{i}\setminus A_{i}^{\left[k\right]}\right\}$ and the assumption in Proposition 6 that $Y⊥A_{i}^{\left(k\right)}|C_{i}^{\left[k\right]}Z_{i}^{\left[k\right]}A_{i}\setminus A_{i}^{\left[k\right]}$ holds. Accordingly, the unique information is preserved removing $A_{i}^{\left(k\right)}$ (Proposition 2). This leads to the inequality $I(Y;C_{i}^{\left[k-1\right]}A_{i}\setminus A_{i}^{\left[k-1\right]}\setminus \setminus Z\setminus Z_{i}^{\left[k-1\right]}|\;Z_{i}^{\left[k-1\right]})\leq$$I(Y;C_{i}^{\left[k\right]}A_{i}\setminus A_{i}^{\left[k\right]}\setminus \setminus Z\setminus Z_{i}^{\left[k\right]}|\;Z_{i}^{\left[k\right]})$. Equation (A3) iterated for $k=1,\cdots ,m_{i}-1$ leads to $I(Y;B_{i}\setminus \setminus Z_{i}^{\left(m_{i}\right)}|Z_{i}^{\left[m_{i}-1\right]})$, with $B_{i}=\left\{C_{i}^{\left[m_{i}-1\right]},A_{i}^{\left(m_{i}\right)}\right\}$. Finally, this unique information by construction is smaller than $I(Y;B_{i}|Z)$. The terms $I(Y;A_{i}\setminus \setminus Z\setminus Z_{i}^{\left(1\right)}|Z_{i}^{\left(1\right)})$ are obtained removing $C_{i}^{\left[1\right]}$ from $I(Y;C_{i}^{\left[k\right]}A_{i}\setminus A_{i}^{\left[k-1\right]}\setminus \setminus Z\setminus Z_{i}^{\left[k\right]}|Z_{i}^{\left[k\right]})$ by monotonicity, from step k=1. □

## Appendix B. Proof of Theorem 2

**Proof of Theorem 2.** The proof proceeds by induction like the proof of Theorem 1 in [27]. To render the notation less heavy, we simplify $an_{G^{\prime }}\left(A_{i}\right)$ to $an(A_{i})$ and $d_{i}\left(G^{\prime };Z\right)$ to $d_{i}$, with both $G^{\prime }$ and Z fixed. Define $V_{Z}=\left\{V_{Z}^{\left(1\right)},\cdots ,V_{Z}^{\left(m\right)}\right\}$, with $V_{Z}^{\left(j\right)}\equiv \left(an\left(Z_{j}\right)\cap an\left(A_{\left[n\right]}\right)\right)\setminus W$. Without loss of generality, for j=1,⋯,m we sequentially apply the chain rule to separate the information that each subset $V_{Z}^{\left(j\right)}$ provides about Y after the chain rule has already been applied to $V_{Z}^{\left[j-1\right]}\equiv \left\{V_{Z}^{\left(1\right)},...,V_{Z}^{\left(j-1\right)}\right\}$. At the j-th iteration, we obtain

(A4) $$I\left(Y;\tilde{an}\left(A_{\left[n\right]}\right)|Z,V_{Z}^{\left[j-1\right]}\right)=I\left(Y;V_{Z}^{\left(j\right)}|Z,V_{Z}^{\left[j-1\right]}\right)+I\left(Y;\tilde{an}\left(A_{\left[n\right]}\right)|Z,V_{Z}^{\left[j\right]}\right).$$

The iterative induction step proceeds as follows. Assume that the inequality of Theorem 2 holds for

(A5) $$I\left(Y;\tilde{an}\left(A_{\left[n\right]}\right)|Z,V_{Z}^{\left[j\right]}\right)\geq \sum_{i=1}^{n}\frac{1}{d_{i}}I\left(Y;\tilde{an}\left(A_{i}\right)|Z,V_{Z}^{\left[j\right]}\right).$$

We show that then the inequality also holds for $I(Y;\tilde{an}\left(A_{\left[n\right]}\right)|Z,V_{Z}^{\left[j-1\right]})$. First, if $V_{Z}^{\left(j\right)}\subseteq \left\{Z,V_{Z}^{\left[j-1\right]}\right\}$ then $\left\{Z,V_{Z}^{\left[j-1\right]}\right\}$$=\{Z,V_{Z}^{\left[j\right]}\}$ and Equation (A5) already provides the desired inequality. We continue with $V_{Z}^{\left(j\right)}⊈\left\{Z,V_{Z}^{\left[j-1\right]}\right\}$. Split the sum in Equation (A5) into two sums, one containing groups in the set $S(G^{\prime };Z_{j})$ (see its definition below Equation (7)), and the other groups not in $S(G^{\prime };Z_{j})$. For the sake of simplifying notation, we use $S_{Z_{j}}$ for $S(G^{\prime };Z_{j})$, given that $G^{\prime }$ is fixed. We first consider the sum of groups in $S_{Z_{j}}$:

(A6) $$\begin{array}{cc} \; & \sum_{A_{i}\in S_{Z_{j}}}\frac{1}{d_{i}}I\left(Y;\tilde{an}\left(A_{i}\right)|Z,V_{Z}^{\left[j\right]}\right)\overset{\left(a\right)}{=}\\ \; & \sum_{A_{i}\in S_{Z_{j}}}\frac{1}{d_{i}}\left[I(Y;\tilde{an}\left(A_{i}\right),V_{Z}^{\left(j\right)}|Z,V_{Z}^{\left[j-1\right]})\right.-\left.I(Y;V_{Z}^{\left(j\right)}|Z,V_{Z}^{\left[j-1\right]})\right]\overset{\left(b\right)}{\geq }\\ & \left[\sum_{A_{i}\in S_{Z_{j}}}\frac{1}{d_{i}}I\left(Y;\tilde{an}\left(A_{i}\right),V_{Z}^{\left(j\right)}|Z,V_{Z}^{\left[j-1\right]}\right)\right]-I\left(Y;V_{Z}^{\left(j\right)}\right)\left|Z,V_{Z}^{\left[j-1\right]}\right)\overset{\left(c\right)}{\geq }\\ \; & \left[\sum_{A_{i}\in S_{Z_{j}}}\frac{1}{d_{i}}I\left(Y;\tilde{an}\left(A_{i}\right)|Z,V_{Z}^{\left[j-1\right]}\right)\right]-I\left(Y;V_{Z}^{\left(j\right)}\right)\left|Z,V_{Z}^{\left[j-1\right]}\right). \end{array}$$

Equality (a) follows from the chain rule. Inequality (b) follows from the definition of $d_{i}\left(G^{\prime };Z\right)$ (in short $d_{i}$) in Equation (8). By construction $d_{i}\left(G^{\prime };Z\right)$ is equal to or higher than all $d_{i}\left(G^{\prime };Z_{j}\right)$ and $d_{i}\left(G^{\prime };Z_{j}\right)$ is the maximal number of groups intersecting together with $an(A_{i};Z_{j})$ (Equation (7)). For any group *i* within $S(G^{\prime };Z_{j})$, $an(A_{i};Z_{j})$ includes $an\left(Z_{j}\right)\cap an\left(A_{\left[n\right]}\right)$ and hence $d_{i}\left(G^{\prime };Z_{j}\right)$$\geq |S(G^{\prime };Z_{j})|$, so that $\sum_{A_{i}\in S_{Z_{j}}}1/d_{i}\leq 1$. Inequality (c) follows from the monotonicity property of the mutual information. For the other sum

(A7) $$\begin{array}{cc} \; & \sum_{A_{i}\notin S_{Z_{j}}}\frac{1}{d_{i}}I\left(Y;\tilde{an}\left(A_{i}\right)|Z,V_{Z}^{\left[j\right]}\right)\overset{\left(a\right)}{\geq }\sum_{A_{i}\notin S_{Z_{j}}}\frac{1}{d_{i}}I(Y;\tilde{an}\left(A_{i}\right)\setminus V_{Z}^{\left(j\right)}\left|Z,V_{Z}^{\left[j-1\right]}\right)\overset{\left(b\right)}{=}\\ & \sum_{A_{i}\notin S_{Z_{j}}}\frac{1}{d_{i}}I\left(Y;\tilde{an}\left(A_{i}\right)|Z,V_{Z}^{\left[j-1\right]}\right). \end{array}$$

Inequality (a) follows from applying Lemma 1 (ii), with $A=\tilde{an}\left(A_{i}\right)\setminus \left\{Z,V_{Z}^{\left[j\right]}\right\}$, $B=\{Z,V_{Z}^{\left[j-1\right]}\}$, and $C=V_{Z}^{\left(j\right)}\setminus \left\{Z,V_{Z}^{\left[j-1\right]}\right\}$. Independence A⊥C|B holds because $A_{i}\notin S\left(G^{\prime };Z_{j}\right)$ means $an\left(A_{i}\right)⊥an\left(Z_{j}\right)\cap an\left(A_{\left[n\right]}\right)|Z$ (Equation (7)), which implies $\tilde{an}\left(A_{i}\right)⊥V_{Z}^{\left(j\right)}|Z$, given that $V_{Z}^{\left(j\right)}\equiv \left(an\left(Z_{j}\right)\cap an\left(A_{\left[n\right]}\right)\right)\setminus W$. Assuming faithfulness, since all the variables in $V_{Z}^{\left[j-1\right]}$ are ancestors of Z, conditioning on $\left\{Z,V_{Z}^{\left[j-1\right]}\right\}$ does not create any new dependence (activating colliders) that did not exist conditioning on Z. Equality (b) holds because given Equation (7) an overlap between $\tilde{an}\left(A_{i}\right)\setminus Z$ and $V_{Z}^{\left(j\right)}\setminus Z$ is in contradiction with $A_{i}\notin S\left(G^{\prime };Z_{j}\right)$. Combining Equations (A6) and (A7) in the r.h.s of Equation (A5), we obtain that

(A8) $$I\left(Y;\tilde{an}\left(A_{\left[n\right]}\right)|Z,V_{Z}^{\left[j\right]}\right)\geq \left[\sum_{i=1}^{n}\frac{1}{d_{i}}I\left(Y;\tilde{an}\left(A_{i}\right)|Z,V_{Z}^{\left[j-1\right]}\right)\right]-I\left(Y;V_{Z}^{\left(j\right)}|Z,V_{Z}^{\left[j-1\right]}\right).$$

We then insert this inequality in Equation (A4) to obtain the final desired inequality:

(A9) $$I\left(Y;\tilde{an}\left(A_{\left[n\right]}\right)|Z,V_{Z}^{\left[j-1\right]}\right)\geq \sum_{i=1}^{n}\frac{1}{d_{i}}I\left(Y;\tilde{an}\left(A_{i}\right)|Z,V_{Z}^{\left[j-1\right]}\right).$$

After subtracting $V_{Z}=\left\{V_{Z}^{\left(1\right)},\cdots ,V_{Z}^{\left(m\right)}\right\}$, the validity of the inequality of Theorem 2 depends on the validity of

(A10) $$I\left(Y;\tilde{an}\left(A_{\left[n\right]}\right)|Z,an\left(Z\right)\cap \tilde{an}\left(A_{\left[n\right]}\right)\right)\geq \sum_{i=1}^{n}\frac{1}{d_{i}}I\left(Y;\tilde{an}\left(A_{i}\right)|Z,an\left(Z\right)\cap \tilde{an}\left(A_{\left[n\right]}\right)\right).$$

At each iterations, if $\tilde{an}\left(A_{\left[n\right]}\right)\setminus \left\{Z,V_{Z}^{\left[j\right]}\right\}$ is empty, the corresponding assumption in Equation (A5) is trivially fulfilled and the proof ends. Otherwise, the proof by induction continues further subtracting variables from $\tilde{an}\left(A_{\left[n\right]}\right)\setminus an\left(Z\right)$. We define the set of groups whose ancestral set in $G^{\prime }$ overlaps with W:

(A11) $$S_{W}\equiv \left\{A_{i}\in A_{\left[n\right]}:an_{G^{\prime }}\left(A_{i}\right)\cap W\neq \emptyset \right\}.$$

We select subsets of variables to be subtracted using the same criterion used in the proof of Theorem 1 of [27], but restricting the groups used as reference in each iteration to be in the complementary set ${\bar{S}}_{W}$, i.e., with $an\left(A_{i}\right)=\tilde{an}\left(A_{i}\right)$. In more detail, consider without loss of generality that in the first iteration the j-th group $A_{j}$ is taken as reference. Define $V^{\left(0\right)}\equiv V_{Z}$, where $V_{Z}=\left\{V_{Z}^{\left(1\right)},...,V_{Z}^{\left(m\right)}\right\}$ has already been subtracted from $\tilde{an}\left(A_{\left[n\right]}\right)$. With $A_{j}$ as reference, find the joint intersection of $\tilde{an}\left(A_{j}\right)\setminus V^{\left(0\right)}$ with a maximal number of other groups $\tilde{an}\left(A_{j^{\prime }}\right)\setminus V^{\left(0\right)}$, $j^{\prime }\neq j$. Define $S_{j}^{\left(1\right)}$ as the set of groups in this intersection. The superindex indicates that this set is associated with the first iteration of this part of the induction procedure, while the subindex indicates that the j-th group is the reference. The subindex will be omitted when the group used as reference is not relevant. Define $V_{j}^{\left(1\right)}\equiv {⋂}_{A_{i}\in S_{j}^{\left(1\right)}}\tilde{an}\left(A_{i}\right)\setminus V^{\left(0\right)}$ as the set of variables contained in this intersection. This subset is subtracted in the first iteration. Analogously, consider that in the k-th iteration $V^{\left[k-1\right]}\equiv \left\{V^{\left(0\right)},...,V^{\left(k-1\right)}\right\}$ has already been subtracted and the j-th group is taken as reference. Then $S_{j}^{\left(k\right)}$ is determined by the joint intersection of $\tilde{an}\left(A_{j}\right)\setminus V^{\left[k-1\right]}$ with a maximal number of other groups $\tilde{an}\left(A_{j^{\prime }}\right)\setminus V^{\left[k-1\right]}$, $j^{\prime }\neq j$. The subset of variables subtracted in the k-th iteration is $V_{j}^{\left(k\right)}\equiv {⋂}_{A_{i}\in S_{j}^{\left(k\right)}}\tilde{an}\left(A_{i}\right)\setminus V^{\left[k-1\right]}$. By construction $V_{j}^{\left(k\right)}\subseteq {\tilde{an}}_{G^{\prime }}\left(A_{\left[n\right]}\right)\setminus V^{\left[k-1\right]}$. Furthermore, $|S_{j}^{\left(k\right)}|\leq d_{j}\left(G^{\prime };Z\right)$, since $d_{j}\left(G^{\prime };Z\right)$ is maximal among $d_{j}\left(G^{\prime };Z_{i}\right)$ for i=1,⋯,m (Equation (8)) for all intersections of the augmented ancestral sets defined in Equation (7), while $S_{j}^{\left(k\right)}$ is determined by only intersections with no support in $V^{\left[k-1\right]}$ and only among the reduced ancestral sets. So far, we have described the selection of subsets to be subtracted. We now look at the iterative induction step when removing a subset $V_{j}^{\left(k\right)}$ after the previous k−1 iterations have already been performed. Consider

(A12) $$\begin{array}{cc} \; & I\left(Y;\tilde{an}\left(A_{\left[n\right]}\right)|Z,V^{\left[k-1\right]}\right)\overset{\left(a\right)}{=}I\left(Y;\tilde{an}\left(A_{\left[n\right]}\right)V_{j}^{\left(k\right)}|Z,V^{\left[k-1\right]}\right)\\ \; & \overset{\left(b\right)}{=}I\left(Y;V_{j}^{\left(k\right)}|Z,V^{\left[k-1\right]}\right)+I\left(Y;\tilde{an}\left(A_{\left[n\right]}\right)|Z,V^{\left[k\right]}\right). \end{array}$$

Equality (a) follows from $V_{j}^{\left(k\right)}\subseteq \tilde{an}\left(A_{\left[n\right]}\right)\setminus V^{\left[k-1\right]}$. Equality (b) is an application of the chain rule. We now show that under the assumption that

(A13) $$I\left(Y;\tilde{an}\left(A_{\left[n\right]}\right)|Z,V^{\left[k\right]}\right)\geq \sum_{i=1}^{n}\frac{1}{d_{i}}I\left(Y;\tilde{an}\left(A_{i}\right)|Z,V^{\left[k\right]}\right),$$

the analogous inequality holds for $I(Y;\tilde{an}\left(A_{\left[n\right]}\right)|Z,V^{\left[k-1\right]})$. We again break down the sum of the groups into two sums, one containing groups in $S_{j}^{\left(k\right)}$ and the other the rest. We first consider the sum of groups in $S_{j}^{\left(k\right)}$:

(A14) $$\begin{array}{cc} \; & \sum_{A_{i}\in S_{j}^{\left(k\right)}}\frac{1}{d_{i}}I\left(Y;\tilde{an}\left(A_{i}\right)|Z,V^{\left[k\right]}\right)\overset{\left(a\right)}{=}\\ \; & \sum_{A_{i}\in S_{j}^{\left(k\right)}}\frac{1}{d_{i}}\left[I(Y;\tilde{an}\left(A_{i}\right)V_{j}^{\left(k\right)}|Z,V^{\left[k-1\right]})\right.-\left.I(Y;V_{j}^{\left(k\right)}|Z,V^{\left[k-1\right]})\right]\overset{\left(b\right)}{\geq }\\ \; & \left[\sum_{A_{i}\in S_{j}^{\left(k\right)}}\frac{1}{d_{i}}I\left(Y;\tilde{an}\left(A_{i}\right)V_{j}^{\left(k\right)}|Z,V^{\left[k-1\right]}\right)\right]-I\left(Y;V_{j}^{\left(k\right)}|Z,V^{\left[k-1\right]}\right)\overset{\left(c\right)}{\geq }\\ \; & \left[\sum_{A_{i}\in S_{j}^{\left(k\right)}}\frac{1}{d_{i}}I\left(Y;\tilde{an}\left(A_{i}\right)|Z,V^{\left[k-1\right]}\right)\right]-I\left(Y;V_{j}^{\left(k\right)}|Z,V^{\left[k-1\right]}\right). \end{array}$$

Equality (a) follows from the chain rule. Inequality (b) holds because $|S_{j}^{\left(k\right)}|\leq d_{i}\left(G^{\prime };Z\right)$ for all $A_{i}\in S_{j}^{\left(k\right)}$. This is because the intersection that determines $S_{j}^{\left(k\right)}$ contains variables from $A_{j}$ and from all other groups $A_{i}\in S_{j}^{\left(k\right)}$, and hence for all these groups it also determines $d_{i}\left(G^{\prime };Z\right)$ unless an intersection with more groups exists for $A_{i}$. Given $|S_{j}^{\left(k\right)}|\leq d_{i}\left(G^{\prime };Z\right)$ for all $A_{i}\in S_{j}^{\left(k\right)}$, it follows that $\sum_{A_{i}\in S_{j}^{\left(k\right)}}1/d_{i}\left(G^{\prime };Z\right)\leq \sum_{A_{i}\in S_{j}^{\left(k\right)}}1/\left|S_{j}^{\left(k\right)}\right|=1$. Inequality (c) follows from monotonicity of mutual information. We now consider the sum involving groups that do not belong to $S_{j}^{\left(k\right)}$:

(A15) $$\sum_{A_{i}\notin S_{j}^{\left(k\right)}}\frac{1}{d_{i}}I\left(Y;\tilde{an}\left(A_{i}\right)|Z,V^{\left[k\right]}\right)\geq \sum_{A_{i}\notin S_{j}^{\left(k\right)}}\frac{1}{d_{i}}I\left(Y;\tilde{an}\left(A_{i}\right)|Z,V^{\left[k-1\right]}\right).$$

The inequality holds applying Lemma 1(ii) with $A=\tilde{an}\left(A_{i}\right)\setminus \left\{Z,V^{\left[k\right]}\right\}$, $B=\{Z,V^{\left[k-1\right]}\}$, and $C=V_{j}^{\left(k\right)}\setminus \left\{Z,V^{\left[k-1\right]}\right\}$. By construction, $V_{j}^{\left(k\right)}\cap \left\{Z,V^{\left[k-1\right]}\right\}=\emptyset$ and hence $C=V_{j}^{\left(k\right)}$. Furthermore, $\tilde{an}\left(A_{i}\right)\setminus \left\{Z,V^{\left[k-1\right]}\right\}$ is equal to $\tilde{an}\left(A_{i}\right)\setminus \left\{Z,V^{\left[k\right]}\right\}$ given that $A_{i}\notin S_{j}^{\left(k\right)}$. An intersection of $\tilde{an}\left(A_{i}\right)\setminus \left\{Z,V^{\left[k-1\right]}\right\}$ and $V_{j}^{\left(k\right)}$ is contradictory with the definition of $V_{j}^{\left(k\right)}$, since $|S_{j}^{\left(k\right)}|$ is determined to be maximal, but would increase to $|S_{j}^{\left(k\right)}|+1$ if defined by that intersection, and that would lead to $A_{i}\in S_{j}^{\left(k\right)}$ instead. Lemma 1(ii) applies given the independence A⊥C|B. We now prove that this independence holds. We proceed discarding the presence of all types of paths in *G* that would create a dependence AC|B. Under the faithfulness assumption, we examine the four different types of paths in *G* that could create a dependence. First, there is a variable $X_{r}\in C$ and a variable $X_{l}\in A$ with an active directed path in *G* from $X_{r}$ to $X_{l}$, not blocked by B. If this path is active in *G* conditioning on $B=\{Z,V^{\left[k-1\right]}\}$, it also exists in any $G^{\prime }=G_{{\underline{Z}}^{\prime }}$, with $Z^{\prime }\subseteq Z$, since the removal of outgoing arrows has the same effect as conditioning for the paths in which the conditioning variables are noncolliders (i.e., do not have two incoming arrows). This active directed path means that $X_{r}$ would be an ancestor of $X_{l}$ in $G^{\prime }$. Therefore, given $X_{l}\in A$ and $X_{r}\in C$, $X_{r}$ itself would be part of ${\tilde{an}}_{G^{\prime }}\left(A_{i}\right)\setminus V^{\left[k-1\right]}$. However, as argued above, an intersection of ${\tilde{an}}_{G^{\prime }}\left(A_{i}\right)\setminus V^{\left[k-1\right]}$ and $V_{j}^{\left(k\right)}$ is contradictory with $A_{i}\notin S_{j}^{\left(k\right)}$. Second, there is a variable $X_{r}\in C$ and a variable $X_{l}\in A$ with an active directed path in *G* from $X_{l}$ to $X_{r}$, not blocked by B. Again, this path being active in *G* when conditioning on $B=\{Z,V^{\left[k-1\right]}\}$, means that it also exists in any $G^{\prime }=G_{{\underline{Z}}^{\prime }}$, with $Z^{\prime }\subseteq Z$. Therefore, $X_{l}$ would be an ancestor of $X_{r}$ in $G^{\prime }$. This is again a contradiction with the definition of $V_{j}^{\left(k\right)}$ because it could be redefined to include $|S_{j}^{\left(k\right)}|+1$ groups, since $X_{l}$ would be an ancestor of all groups intersecting in $V_{j}^{\left(k\right)}$. Third, there is a variable $X_{r}\in C$, a variable $X_{l}\in A$, and another variable $X_{h}$ that is not part of A nor C with an active directed path in *G* from $X_{h}$ to $X_{r}$ and an active directed path from $X_{h}$ to $X_{l}$, both not blocked by B. This would also imply that these directed paths exist in $G^{\prime }=G_{{\underline{Z}}^{\prime }}$, with $Z^{\prime }\subseteq Z$, and hence $X_{h}$ is an ancestor of A and C in $G^{\prime }$. Since $X_{h}$ is an ancestor of $A=\tilde{an}\left(A_{i}\right)\setminus \left\{Z,V^{\left[k-1\right]}\right\}$ but by construction $X_{h}\notin A$, this means that $X_{h}$ has to be part of $\left\{Z,V^{\left[k-1\right]}\right\}$ or of W, since any ancestor of $an(A_{i})$ is part of $an(A_{i})$. If $X_{h}\in \left\{Z,V^{\left[k-1\right]}\right\}$, conditioning on $B=\{Z,V^{\left[k-1\right]}\}$ would prevent from having active directed paths from $X_{h}$ to $X_{r}$ and from $X_{h}$ to $X_{l}$, leading to a contradiction. We now consider the case $X_{h}\in W$. Since $X_{h}$ is an ancestor of $C=V_{j}^{\left(k\right)}$, by construction of $V_{j}^{\left(k\right)}$, $X_{h}$ is an ancestor of $\tilde{an}\left(A_{j}\right)\setminus \left\{Z,V^{\left[k-1\right]}\right\}$. This means that $an(A_{j})$ includes $X_{h}\in W$ which, given Equation (A11), is in contradiction with the criterion for selection of reference groups such that $A_{j}\in {\bar{S}}_{W}$. In these three types of cases, an active path would exist despite conditioning on B. In the last type, a path would be activated by conditioning on B. At least one variable $X_{h}\in B=\left\{Z,V^{\left[k-1\right]}\right\}$ has to be a collider or a descendant of a collider along the path that conditioning activates. Consider first that a single collider $X_{h}$ is involved. For the collider to activate the path, it must exist an active directed subpath to $X_{h}$ from a variable $X_{r}$ that is part of C or part of its ancestor set in $G^{\prime }$. Since this directed subpath is active in *G* when conditioning on B, it is also active in $G^{\prime }$. This means that $X_{r}$ would be an ancestor of $X_{h}$ in $G^{\prime }$. If $X_{h}$ is part of Z or part of $V^{\left(0\right)}\equiv \left(an_{G^{\prime }}\left(Z\right)\cap an_{G^{\prime }}\left(A_{\left[n\right]}\right)\right)\setminus W$, then $X_{r}$ being an ancestor of $X_{h}$ means that it is part of $an_{G^{\prime }}\left(Z\right)\cap an_{G^{\prime }}\left(A_{\left[n\right]}\right)$. Accordingly, by definition of $V^{\left(0\right)}$, $X_{r}$ would be part of $V^{\left(0\right)}$ or of W. The former option leads to a contradiction because $V^{\left(0\right)}$ has already been removed from $\tilde{an}\left(A_{\left[n\right]}\right)$ and is part of the conditioning variables, so that the subpath from $X_{r}$ to $X_{h}$ could not be part of the path activated by conditioning on the collider. The latter option, $X_{r}$ being part of W, is in contradiction with it being an ancestor of $C=V_{j}^{\left(k\right)}$, since this means being an ancestor of the group $A_{j}$ taken as reference to build $V_{j}^{\left(k\right)}$, which by construction is chosen from ${\bar{S}}_{W}$. We continue considering that $X_{h}$ is part of $V^{\left(k^{\prime }\right)}\in V^{\left[k-1\right]}$, for $0<k^{\prime }\leq k-1$. In this case, $X_{r}$ being an ancestor of $X_{h}$ would mean that either $X_{r}$ is in W or it would have been possible to define $V^{\left(k^{\prime }\right)}$ to include $X_{r}$. In the former case, this leads to a contradiction because for $0<k^{\prime }\leq k-1$ all $V^{\left(k^{\prime }\right)}$ have been constructed taking as reference a group belonging to ${\bar{S}}_{W}$. In the latter case, this leads to a contradiction because $V^{\left(k^{\prime }\right)}$ is constructed to include all variables in the intersection with the maximum number of groups. The same reasoning holds if the activated path contains more than one collider from B, by selecting the collider $X_{h}$ closest to a variable in C along the path. Since for all four types of paths that could lead to AC|B we reach a contradiction, A⊥C|B holds and Lemma 1(ii) can be applied to obtain the inequality in Equation (A15). Combining Equations (A14) and (A15) with the r.h.s. of Equation (A13), we obtain that

(A16) $$I\left(Y;\tilde{an}\left(A_{\left[n\right]}\right)|Z,V^{\left[k\right]}\right)\geq \left[\sum_{i=1}^{n}\frac{1}{d_{i}}I\left(Y;\tilde{an}\left(A_{i}\right)|Z,V^{\left[k-1\right]}\right)\right]-I\left(Y;V_{j}^{\left(k\right)}|Z,V^{\left[k-1\right]}\right).$$

We then insert this inequality in Equation (A12) to obtain the desired inequality:

(A17) $$I\left(Y;\tilde{an}\left(A_{\left[n\right]}\right)|Z,V^{\left[k-1\right]}\right)\geq \sum_{i=1}^{n}\frac{1}{d_{i}}I\left(Y;\tilde{an}\left(A_{i}\right)|Z,V^{\left[k-1\right]}\right).$$

After the completion of these iterations, all variables in $(an\left(Z\right)\cap an\left(A_{\left[n\right]}\right))\setminus W$ and in groups from ${\bar{S}}_{W}$ have been subtracted from $\tilde{an}\left(A_{\left[n\right]}\right)$. The proof ends if after some iteration $\tilde{an}\left(A_{\left[n\right]}\right)\setminus V^{\left[k\right]}$ is empty. In particular, the proof ends if W is empty and hence all groups are already subtracted. Otherwise, assume that $m^{\prime }$ iterations have been carried out when finishing this step. The proof by induction continues with a single additional step for the remaining groups $S_{W}$. Select a single variable $X_{0}$ out of $\tilde{an}\left(A_{\left[n\right]}\right)\setminus V^{\left[m^{\prime }\right]}$ that is only contained in groups in $S_{W}$ and apply the chain rule

(A18) $$\begin{array}{cc} \; & I\left(Y;\tilde{an}\left(A_{\left[n\right]}\right)|Z,V^{\left[m^{\prime }\right]}\right)=I(Y;\tilde{an}\left(A_{\left[n\right]}\right)\setminus X_{0}|Z,V^{\left[m^{\prime }\right]})+I(Y;X_{0}|Z,\tilde{an}\left(A_{\left[n\right]}\right)\setminus X_{0})=\\ \; & I(Y;\tilde{an}\left(A_{\left[n\right]}\right)\setminus X_{0}|Z,V^{\left[m^{\prime }\right]})+I(Y;\tilde{an}\left(A_{\left[n\right]}\right)|Z,\tilde{an}\left(A_{\left[n\right]}\right)\setminus X_{0}). \end{array}$$

The iterative induction step should prove that if the inequality of the theorem holds for $I(Y;\tilde{an}\left(A_{\left[n\right]}\right)|Z,\tilde{an}\left(A_{\left[n\right]}\right)\setminus X_{0})$ it is also true for $I(Y;\tilde{an}\left(A_{\left[n\right]}\right)|Z,V^{\left[m^{\prime }\right]})$. We will prove this below. Before we show that the inequality

(A19) $$I(Y;\tilde{an}\left(A_{\left[n\right]}\right)|Z,\tilde{an}\left(A_{\left[n\right]}\right)\setminus X_{0})\geq \sum_{i=1}^{n}\frac{1}{d_{i}}I(Y;\tilde{an}\left(A_{i}\right)|Z,\tilde{an}\left(A_{\left[n\right]}\right)\setminus X_{0})$$

always holds, and hence it provides the base case for the induction proof. The base case is true because

(A20) $$\begin{array}{cc} \; & \sum_{i=1}^{n}\frac{1}{d_{i}}I(Y;\tilde{an}\left(A_{i}\right)|Z,\tilde{an}\left(A_{\left[n\right]}\right)\setminus X_{0})\overset{\left(a\right)}{=}\sum_{i:X_{0}\in \tilde{an}\left(A_{i}\right)}\frac{1}{d_{i}}I(Y;X_{0}|Z,\tilde{an}\left(A_{\left[n\right]}\right)\setminus X_{0})\\ \; & \overset{\left(b\right)}{=}I(Y;X_{0}|Z,\tilde{an}\left(A_{\left[n\right]}\right)\setminus X_{0})\left[\sum_{i:X_{0}\in \tilde{an}\left(A_{i}\right)}\frac{1}{d_{i}}\right]\overset{\left(c\right)}{\leq }I(Y;X_{0}|Z,\tilde{an}\left(A_{\left[n\right]}\right)\setminus X_{0}). \end{array}$$

Equality (a) holds because $I(Y;\tilde{an}\left(A_{i}\right)|Z,\tilde{an}\left(A_{\left[n\right]}\right)\setminus X_{0})$ is zero for the terms that do not contain $X_{0}$. Equality (b) holds because the information term is the same across the sum and can be factorized. Inequality (c) is justified as follows. Let $N_{0}$ be the number of groups that contain $X_{0}$, and hence that intersect in $X_{0}$. For these groups, $d_{i}\left(G^{\prime };Z\right)$ is higher than or equal to $N_{0}$. This means that $\sum_{i:X_{0}\in \tilde{an}\left(A_{i}\right)}1/d_{i}\left(G^{\prime };Z\right)\leq 1$. We now complete the proof of the last iterative induction step:

(A21) $$\begin{array}{cc} \; & \sum_{i=1}^{n}\frac{1}{d_{i}}I(Y;\tilde{an}\left(A_{i}\right)|Z,\tilde{an}\left(A_{\left[n\right]}\right)\setminus X_{0})\overset{\left(a\right)}{=}\sum_{A_{i}\in S_{W}}\frac{1}{d_{i}}I(Y;\tilde{an}\left(A_{i}\right)|Z,\tilde{an}\left(A_{\left[n\right]}\right)\setminus X_{0})\overset{\left(b\right)}{=}\\ \; & \sum_{A_{i}\in S_{W}}\frac{1}{d_{i}}\left[I(Y;\tilde{an}\left(A_{i}\right),\tilde{an}\left(A_{\left[n\right]}\right)\setminus X_{0}|Z,V^{\left[m^{\prime }\right]})\right.-\left.I(Y;\tilde{an}\left(A_{\left[n\right]}\right)\setminus X_{0}|Z,V^{\left[m^{\prime }\right]})\right]\overset{\left(c\right)}{\geq }\\ \; & \left[\sum_{A_{i}\in S_{W}}\frac{1}{d_{i}}I(Y;\tilde{an}\left(A_{i}\right),\tilde{an}\left(A_{\left[n\right]}\right)\setminus X_{0}\left|Z,V^{\left[m^{\prime }\right]}\right)\right]-I(Y;\tilde{an}\left(A_{\left[n\right]}\right)\setminus X_{0}\left|Z,V^{\left[m^{\prime }\right]}\right)\overset{\left(d\right)}{\geq }\\ \; & \left[\sum_{A_{i}\in S_{W}}\frac{1}{d_{i}}I\left(Y;\tilde{an}\left(A_{i}\right)|Z,V^{\left[m^{\prime }\right]}\right)\right]-I(Y;\tilde{an}\left(A_{\left[n\right]}\right)\setminus X_{0}\left|Z,V^{\left[m^{\prime }\right]}\right)\overset{\left(e\right)}{=}\\ \; & \left[\sum_{i=1}^{n}\frac{1}{d_{i}}I\left(Y;\tilde{an}\left(A_{i}\right)|Z,V^{\left[m^{\prime }\right]}\right)\right]-I(Y;\tilde{an}\left(A_{\left[n\right]}\right)\setminus X_{0}\left|Z,V^{\left[m^{\prime }\right]}\right). \end{array}$$

Equality (a) holds because $X_{0}$ is selected to be contained only in groups in $S_{W}$. Equality (b) follows from the chain rule and from $V^{\left[m^{\prime }\right]}\subseteq \tilde{an}\left(A_{\left[n\right]}\right)\setminus X_{0}$. Inequality (c) holds because, for all $A_{i}\in S_{W}$, $d_{i}\left(G^{\prime };Z\right)$ is higher than or equal to $|S_{W}|$, since their ancestral sets intersect at $W_{0}$, which is an ancestor of all variables in W. This means that $\sum_{A_{i}\in S_{W}}1/d_{i}\left(G^{\prime };Z\right)\leq 1$. Inequality (d) follows from the monotonicity of mutual information, and equality (e) holds because $\tilde{an}\left(A_{i}\right)\subseteq \left\{Z,V^{\left[m^{\prime }\right]}\right\}$ for all $A_{i}\notin S_{W}$. We use the last expression in Equation (A21) at the r.h.s of Equation (A19), and combine it with Equation (A18) to obtain

(A22) $$I\left(Y;\tilde{an}\left(A_{\left[n\right]}\right)|Z,V^{\left[m^{\prime }\right]}\right)\geq \sum_{i=1}^{n}\frac{1}{d_{i}}I\left(Y;\tilde{an}\left(A_{i}\right)|Z,V^{\left[m^{\prime }\right]}\right).$$

This completes the iterative induction step of the proof. Since the validity of the base case has also been proven, this completes the proof. □

## Appendix C. On Required Assumptions Relating Independencies and d-Separation

In this Section, we discuss more closely the requirements on the relation between graphical d-separation and statistical independencies needed for the applicability of the derived inequality constraints. As indicated in Section 2.3, so far we have invoked the faithfulness assumption [1,2] in order to simplify the presentation, that is, we have not distinguished between $X{⊥}_{P}Y|S$ and $X{⊥}_{G}Y|S$. We will now make this distinction and reconsider all cases of the proofs of Appendix A and Appendix B where faithfulness has been invoked, showing that in fact it is only required to assume that d-separation is a sufficient condition for statistical independence.

We start with the role that the assumption of d-separation implying independence has in the proof of Propositions 1 and 3. As discussed in Section 1, we envisage the implementation of the tests such that conditional independence requirements of Proposition 1 or 3 are verified in terms of graphical separability for the hypothesized causal structure. In particular, a test from Proposition 1 is to be applied when verifying that for the selected collection and groups it holds that $A_{i}{⊥}_{G}A_{j}\setminus A_{i}|Z\;\forall i,j$. It is then assumed that this implies $A_{i}{⊥}_{P}A_{j}\setminus A_{i}|Z\;\forall i,j$. In the proof of Proposition 1, in step (e) of Equation (A1), Lemma 1(ii) has been applied invoking faithfulness to guarantee that for $X_{k}\in A_{i}$, independencies $X_{k}{⊥}_{P}\left(X_{\left[k-1\right]}\cap A_{j}\right)\setminus A_{i}|\left(X_{\left[k-1\right]}\cap A_{i}\right),Z$, ∀j≠i imply the independence $X_{k}{⊥}_{P}X_{\left[k-1\right]}\setminus A_{i}|\left(X_{\left[k-1\right]}\cap A_{i}\right),Z$. However, while this implication needs to be assumed at the level of independencies, at the level of graphical separability, $X_{k}{⊥}_{G}\left(X_{\left[k-1\right]}\cap A_{j}\right)\setminus A_{i}|\left(X_{\left[k-1\right]}\cap A_{i}\right),Z$, ∀j≠i straightforwardly implies the joint separability $X_{k}{⊥}_{G}X_{\left[k-1\right]}\setminus A_{i}|\left(X_{\left[k-1\right]}\cap A_{i}\right),Z$. This is because the separability of $X_{\left[k-1\right]}\setminus A_{i}$ follows from the lack of active paths for each of the nodes it contains, and hence is equivalent to the separability of $\left(X_{\left[k-1\right]}\cap A_{j}\right)\setminus A_{i}$ for all *j*, which jointly comprise the same nodes. The assumption that d-separation implies independence guarantees the independence $X_{k}{⊥}_{P}X_{\left[k-1\right]}\setminus A_{i}|\left(X_{\left[k-1\right]}\cap A_{i}\right),Z$ from $X_{k}{⊥}_{G}X_{\left[k-1\right]}\setminus A_{i}|\left(X_{\left[k-1\right]}\cap A_{i}\right),Z$, without the need to more broadly require faithfulness. The proof of Proposition 3 relies on an analogous way on the assumption that d-separation implies independence, using it to guarantee the conditional independencies involving the subsets in $B_{\left[n\right]}^{\left(1\right)}$ and $B_{\left[n\right]}^{\left(2\right)}$. In step (d) of Equation (A2), the fact that separability for a joint set of nodes is straightforwardly guaranteed by the separability of each of its nodes is again applied and then mapped to the existence of an independence using this assumption. The fact that conditions $B_{i}^{\left(1\right)}{⊥}_{G}B_{j}^{\left(1\right)}\setminus B_{i}^{\left(1\right)}|Z$ and $B_{i}^{\left(2\right)}{⊥}_{G}B_{j}\setminus B_{i}^{\left(2\right)}|B_{i}^{\left(1\right)}Z$∀i,j can be verified using d-separation instead of estimating independencies from data is crucial in the case that the groups include hidden variables, which precludes the direct evaluation of these independencies.

The next result whose derivation relies on the assumption that d-separation implies independence is Theorem 2. In step (a) of Equation (A7), faithfulness was invoked to guarantee that conditioning on some ancestors of Z cannot create new dependencies that were not already created by conditioning on Z itself. In more detail, it was assumed that if the independence $\tilde{an}\left(A_{i}\right){⊥}_{P}V_{Z}^{\left(j\right)}|Z$ holds then also $\tilde{an}\left(A_{i}\right){⊥}_{P}V_{Z}^{\left(j\right)}|\left\{Z,V_{Z}^{\left[j-1\right]}\right\}$ holds, where $V_{Z}^{\left[j-1\right]}$ are by construction ancestors of Z. Again, at the level of graphical separability this implication is straightforward and does not require any assumption. This is because by definition of d-separation a path is activated both when conditioning on a collider or on any descendant of the collider, and $V_{Z}^{\left[j-1\right]}$ being ancestors of Z means that Z contains a descendant for each node in $V_{Z}^{\left[j-1\right]}$. Accordingly, no assumption is needed to ensure $\tilde{an}\left(A_{i}\right){⊥}_{G}V_{Z}^{\left(j\right)}|\left\{Z,V_{Z}^{\left[j-1\right]}\right\}$ from $\tilde{an}\left(A_{i}\right){⊥}_{G}V_{Z}^{\left(j\right)}|Z$. The assumption that d-separation implies independence is then used to ensure $\tilde{an}\left(A_{i}\right){⊥}_{P}V_{Z}^{\left(j\right)}|\left\{Z,V_{Z}^{\left[j-1\right]}\right\}$ from $\tilde{an}\left(A_{i}\right){⊥}_{G}V_{Z}^{\left(j\right)}|\left\{Z,V_{Z}^{\left[j-1\right]}\right\}$. Faithfulness is also invoked in the proof of Theorem 2 to justify the application of Lemma 1(ii) in Equation (A15). In this case, the existence of an independence $A{⊥}_{P}C|B$ is directly justified in terms of the nonexistence of active paths in the graph, hence guaranteeing $A{⊥}_{G}C|B$ and subsequently using the assumption that d-separation implies independence to derive $A{⊥}_{P}C|B$.

The considerations above show that the assumption that d-separation implies statistical independence is enough to derive the existence of groups-decomposition inequalities under the conditions of Propositions 1 and 3, and of Theorem 2. Furthermore, if unfaithful independencies are present in the data that do not follow from the causal structure, this may decrease the power to reject causal structures testing the inequalities, but will not lead to incorrect rejections. This differs from the impact of unfaithful independencies on the inference of the Markov equivalence class from data [1,2]. In that case, unfaithful independencies can lead to an incorrect reconstruction of the skeleton of the graph or result in contradictory rules for edge orientation. The assumption that d-separation implies statistical independence is substantially weaker than the reverse assumption also included in the faithfulness assumption, namely that statistical independence implies d-separation. The X-OR logic gate is an example that the latter assumption can be violated. Conversely, if the causal graph is meant to reflect the underlying structure of actual physical mechanisms involved in generating the variables, all statistical dependencies need to originate from some paths of influence between the variables. Accordingly, a d-separation that does not lead to an independence can be taken as an indicator that some structure is missing in the causal graph, namely associated with the paths that create the observed dependence. In this regard, it is appropriate to reject a causal structure if it does not fulfill an inequality constraint because graphical separability is not reflected in the corresponding independencies found in the data.
